# Consumer attitudes and misperceptions associated with trends in self-reported cereal foods consumption: cross-sectional study of Western Australian adults, 1995 to 2012

**DOI:** 10.1186/s12889-017-4511-5

**Published:** 2017-06-26

**Authors:** Christina Mary Pollard, Claire Elizabeth Pulker, Xingqiong Meng, Jane Anne Scott, Felicity Claire Denham, Vicky Anne Solah, Deborah Anne Kerr

**Affiliations:** 1 School of Public Health, Curtin University, GPO Box U1987, Perth, Western 6845 Australia; 2 Department of Health in Western Australia, 189 Royal Street, East Perth, New Orleans, Western 6004 Australia; 3 School of Medicine, Flinders University, Sturt Rd, Bedford Park, South 5042 Australia; 4 ARC Training Centre for Innovative Horticultural Products, Tasmanian Institute of Agriculture, School of Land and Food, University of Tasmania, Private Bay 98, Hobart, TAS 7001 Australia

**Keywords:** Grains, Cereal foods, Whole-grain, Bread, Rice, Pasta, Breakfast cereal, Consumption

## Abstract

**Background:**

The reasons for low adherence to cereal dietary guidelines are not well understood but may be related to knowledge, attitudes, beliefs and perceived barriers. This study aims to assess trends in cereal foods consumption, intention to change and factors associated with intake among Western Australian (WA) adults 18 to 64 years.

**Method:**

Cross-sectional data from the 1995, 1998, 2001, 2004, 2009, and 2012 Nutrition Monitoring Survey Series involving 7044 adults were pooled. Outcome variables: types and amount of cereals (bread, rice, pasta, and breakfast cereal) eaten the day prior. Attitudes, knowledge, intentions, weight status and sociodemographic characteristics were measured. Descriptive statistics, multiple binary logistic and multinomial logistic regressions assess factors associated with consumption.

**Results:**

Bread (78%) was the most commonly consumed cereal food. The proportion eating bread decreased across survey years (Odds Ratio OR = 0.31; 95% Confidence Interval; 0.24–0.40 in 2012 versus 1995), as did the amount (4.1 slices of bread in 1995 to 2.4 in 2012). The odds of consuming whole-grain cereal foods increased since 2009 (OR = 1.27; 1.02–1.58 versus 1995 *p* < 0.05). The likelihood of trying to eat less cereal food in the past year was greater in 2012 compared to 1995 (Relative Risk Ratio RRR 10.88; 6.81–17.4). Knowledge of cereal recommendations decreased over time (OR = 0.20; 0.15–0.27 in 2012 versus 1995 *p* < 0.001). Overweight and obese respondents were more likely than healthy weight respondents to have tried to eat less cereals (RRR 1.65; 1.22–2.24 and 1.88; 1.35–2.63 respectively). ‘I already eat enough’ was the main barrier (75% in 1995 to 84% in 2012 (*p* < 0.001)).

**Conclusions:**

WA adults are actively reducing the amount of cereal foods they eat and intake is associated with a misperception of adequacy of intake. Nutrition intervention is needed to increase awareness of the health benefits of cereal foods, particularly whole-grains, and to address barriers to incorporating them daily.

**Trial registration:**

Not applicable.

**Electronic supplementary material:**

The online version of this article (doi:10.1186/s12889-017-4511-5) contains supplementary material, which is available to authorized users.

## Background

Consuming diets high in cereal foods, particularly whole-grains, has been shown to be protective against cardiovascular disease, cancer, Type 2 diabetes, and excess body weight [1–4]. Countries are encouraged to develop, promote and monitor culturally specific food-based dietary guidelines to promote health and prevent or control non-communicable diseases [5]. Australian Dietary Guidelines (ADG) recommend that adults eat plenty of cereal foods, preferably whole-grain [6–8]. The Australian Guide to Healthy Eating, the food selection guide, recommended adults eat at least four standard servings, equivalent to about 500 kJ, of cereal foods per day, particularly whole-grains and/or high cereal fibre varieties [8]. Most adults do not adhere to the cereal ADG and there is emerging evidence from national survey data that intake is declining. Only 7 % of 18,226 women from the Australian Longitudinal Study on Women’s Health met the recommendation [9]. The 2011–2012 Australian Health Survey found in adults aged 19 years and older, the mean intake of cereals foods was less than in the 1995 National Nutrition Survey (164 g (g) compared to 215 g) [10, 11].

Although dietary guidelines of most countries recommend whole-grain foods there is no universally accepted definition [12] making country comparisons challenging. Cereals, also known as grain foods [13], include breads, breakfast cereals, grains, and other products. Since 1981, ADGs direct consumers to choose brown or wholegrain varieties over the ‘regular’ or refined versions (for example cereal germ and bran, either as a whole product such as brown rice, or wholemeal products where the whole-grain has been ground into flour such as that in wholemeal pasta), while excluding energy dense refined cereal foods with added sugars, saturated fats or salt [8, 14].

Australian adults consumed on average 83 g of whole-grain foods and 188 g of refined grains in 2011–2012 and need to eat 160% more whole-grains and 30% less refined grains to meet the cereal recommendation [8].

Previous research shows that people consuming whole-grains tend to have healthier lifestyles overall, and eat more fruit and vegetables [(15, 16], less fat [17, 18], and a more nutrient dense diet [16]. They are also more likely to be physically active [17, 19], non-smokers [15, 17, 19], and have a lower body mass index (BMI) [15, 20]. Those who consume whole-grains are more likely to be older [19, 21], from high socio-economic groups [19, 21], more educated [22], and live in cities [22].

Barriers to eating enough cereal foods, particularly whole-grains, include difficulty identifying them, high prices, perceptions of inferior taste compared to refined grains, poor food preparation skills, and lack of awareness of dietary recommendations [23] and their importance [24]. Monitoring the population’s response to dietary recommendations, including the barriers and promoters to consumption including their beliefs and intentions to change [25, 26] is important to inform the development of policy and interventions to encourage healthful eating.

In Australia however, population surveillance of dietary intake is limited and sporadic with the two most recent national dietary surveys conducted seventeen years apart, in 1995 and 2011–2012. The Western Australian (WA) Department of Health conducts state-wide triennial surveys to monitor changes in self-reported knowledge, attitudes, beliefs and behaviours relating to the ADG. The aim of this paper is to explore trends in consumption of cereal foods intention to change and factors associated with intake among Western Australian adults 18 to 64 years.

## Methods

### Sample

The Nutrition Monitoring Survey Series (NMSS) Computer-Assisted Telephone Interviews of adults aged 18 to 64 years were conducted between July and August in 1995, 1998, 2001, 2004, 2009 and 2012. The 1995 sample was stratified by geographic area and the 1998, 2001, and 2004 samples were quota sampled by gender and geographic area. Telephone numbers were randomly selected by a computer generated random digit dialling program. In 2009 and 2012, samples were randomly drawn from the 2008 and 2011 Electronic White Pages for Western Australia, and stratified according to area of residence. Ethics approval was granted by the WA Department of Health and Curtin University Human Research Ethics Committees. Prior consent was sought from survey participants in all survey years.

### Outcome measures

The type and amount of cereal foods (bread and bread substitutes, rice, pasta, and breakfast cereal) eaten on the day prior to the survey and whether they were whole-grain or regular versions are the key outcomes. For bread, respondents were asked “*How many slices of [bread], [bread rolls], [muffins], [flat bread], [crumpets], [scones], [rice cakes], [small crackers], [large crackers], [damper] or [other bread substitute] did you eat yesterday?*” then, “*What type of bread or bread rolls did you eat yesterday? Was it brown, wholemeal, multigrain, whole-grain, white, white with extra fibre, or another type?”* Respondents were also asked about rice and pasta, *“How many cups of cooked [rice], [pasta] and [spaghetti] did you eat yesterday?”* and *“What type of rice, pasta or spaghetti did you eat? Was it brown, wholemeal?” and* breakfast cereals, *“How many different types of breakfast cereal did you eat yesterday?”* then *“How many cups of breakfast cereal did you eat?”*

Factors potentially associated with cereal consumption (i.e. knowledge, perceptions, barriers, enablers, and stages of change [26] were measured. Respondents were asked *“What do you think is the recommended minimum number of serves of cereal foods that should be eaten each day? One serve of cereal food is equal to one slice of bread, one cup of breakfast cereal, or half a cup of cooked rice or pasta.”*, about the level of attention paid to the health aspects of the food they eat, and actions taken control weight over the last year. Stages of dietary change [26] were measured by: “*In the past 12 months, have you tried to change the total amount of cereal foods such as breakfast cereal, pasta, rice, bread, or bread products that you eat?”* with eat [more, less, or about the same] responses; and “*Do you feel you eat ‘more’, ‘less’, or ‘about the same’ amount of cereal foods in total than you did 12 months ago?*”

Motivators, barriers and promoters were sought using two open ended questions, *“What influences you to try to change the amount of cereal foods you eat?”* and *“What are the main things that make it difficult for you to eat more cereal foods?”*

BMI was derived from self-reported height and weight, adjusted prior to calculation of BMI to account for potential reporting bias [27]. BMI classifications used were overweight (25–29.9 kg/m^2^), obese (≥ 30 kg/m^2^) and healthy weight (< 25 kg/m^2^) [28]. Sociodemographic information (age, gender, highest education level, household income, employment status, country of birth, area of residence, and living arrangements) were also collected. The 2012 NMSS questionnaire is provided in the Additional file 1 as an example of the current survey instrument.

### Statistical analysis

Sampling methods were chosen to represent the WA population. Data were pooled and weighted to account for sampling design and adjusted for gender, age and areas of residence to a single standard population to allow for comparisons over time. Post-estimation adjustment was used to correct for under or over representation using the 2011 Estimated Resident Population for WA aged 18–64 years (the year of the most recent census at the time of analysis) [29]. The survey design setting in Stata was used in the following way: svyset _n [pweight = probselect], poststrata(stdallyrs) postweight(pop2011) vce(linearized) singleunit(missing). The “probselect” refers to the pooled probability of sample selection, and “pop2011” refers to the 2–11 Estimated Resident Population for WA 18–64 years.

Descriptive statistics report the prevalence of cereal food consumption by survey year. Multiple binary and multinomial logistic regressions were used to assess factors associated with each of the cereal food type measured. Covariates in the full model include: gender, age group, education, household income, employment status, whether living with children, country of birth, area of residence (metropolitan versus non-metropolitan), BMI category, attention paid to diet, and attempts to lose weight. Only variables with *p* value <.05 were retained in the final model and reported, with the exception of survey year which was retained in the model regardless of its significance.

All analyses were performed using the survey module of Stata software version 12.0 (StataCorp LP, College Station, TX).

## Results

Sample characteristics of the 7044 adults presented in Table 1 shows the sample characteristics and Table 2 shows consumption patterns. Across all survey years, 78% of respondents said they ate bread and 50% ate breakfast cereal the day prior to the survey. In 2012, 37.2% of respondents ate no whole-grain cereal foods compared with 51.1% in 1995 (*p* < .001).

**Table 1:** Respondent demographics of Western Australian Nutrition Monitoring Survey Series (NMSS)

	1995	1998	2001	2004	2009	2012	Total % ^a^
	*n* = 1002	*n* = 1004	*n* = 1004	*n* = 1202	*n* = 1284	*n* = 1548	*n* = 7044
Sex							
Female	631	502	502	601	830	1005	49.2
Male	371	502	502	601	454	543	50.8
Age group							
18–24 years	119	110	118	103	71	66	15.8
25–34 years	257	210	245	232	180	144	23.0
35-44 years	291	305	296	333	340	377	22.4
45–54 years	207	234	212	297	356	466	21.3
55–64 years	128	145	133	237	337	495	17.4
Area of residence							
Metropolitan	748	751	754	601	965	1011	78.3
Rural areas ^b^	203	190	18	451	290	455	16.9
Remote areas ^b^	51	63	62	150	29	82	4.8
Education							
Less than high school	376	336	303	330	221	211	19.7
High school	251	237	265	257	178	198	21.0
Trade/Certificate/Diploma	90	95	77	177	481	632	25.5
University degree	284	336	344	435	399	504	33.6
Missing	1	0	15	3	5	3	0.3
Employment status							
Currently not in paid employment	330	263	278	285	364	408	26.8
Currently in paid employment	669	741	726	917	920	1139	73.1
Missing	3	0	0	0	0	1	0
Country of birth							
Australia	656	665	668	868	867	1122	68.6
UK/Ireland	189	209	193	155	202	221	15.0
Other countries	157	130	143	179	214	205	16.4
Missing	0	0	0	0	1	0	0

^a^Percentages were weighted for probability of selection and adjusted by age, sex and geographic area to the 2011 Estimated Resident Population of Western Australia

^b^Rural areas have populations between 10,000 and 100,000. Remote areas have populations of less than 10,000. Rural and remote areas were combined for the analysis

**Table 2:** Cereal foods consumed by Western Australian adults, Western Australian Nutrition Monitoring Survey Series (NMSS)

	1995 % [CI]	1998 % [CI]	2001 % [CI]	2004 % [CI]	2009 % [CI]	2012 % [CI]	*p* value^b^
*Proportion consuming*							
Bread *(n = 7040)*	88.4 [86.1,90.3]	87.2 [84.7,89.4]	82.9 [80.2,85.3]	78.5 [75.6,81.2]	75.9 [72.8,78.7]	70.4 [67.4,73.2]	<.001
Breakfast cereal (*n = 7043*)	51.9 [48.4,55.3]	46.7 [43.4,50.0]	48 [44.6,51.4]	50.5 [47.1,53.9]	50.6 [47.3,53.9]	51.2 [48.0,54.3]	.36
Rice (*n = 7037*)	22.7 [19.8,25.8]	21.3 [18.7,24.2]	22.0 [19.3,24.9]	25.2 [22.3,28.4]	27.5 [24.5,30.7]	25.7 [23.1,28.6]	.02
Bread substitute (*n = 7042*)	21.6 [18.9,24.5]	21.1 [18.5,23.8]	20.4 [17.9,23.1]	24.1 [21.4,27.1]	21.7 [19.2,24.5]	21.7 [19.1,24.5]	.64
Pasta (*n = 7038*)	19 [16.5,21.7]	22.5 [19.9,25.5]	25.2 [22.3,28.3]	22.1 [19.4,25.1]	20.3 [17.8,23.0]	19.1 [16.7,21.8]	.03
*Main type of bread (consumers only, n = 5589)*							<.001
White	42.4 [38.8,46.1]	46.9 [43.3,50.4]	58 [54.4,61.5]	47.9 [44.1,51.7]	30.3 [27.0,33.9]	31.5 [28.1,35.1]	
Brown or wholemeal	28.9 [25.7,32.3]	23.4 [20.7,26.4]	15.7 [13.4,18.3]	22.9 [19.9,26.2]	26.4 [23.3,29.8]	24.2 [21.1,27.6]	
Multigrain or whole-grain	17.1 [14.5,20.0]	18.6 [16.1,21.4]	11.9 [9.8,14.4]	19.3 [16.5,22.4]	36.7 [33.1,40.4]	35.1 [31.6,38.8]	
White with extra fibre	8.8 [7.0,11.1]	8.7 [6.9,10.9]	8.4 [6.6,10.7]	5.6 [4.0,7.8]	2.7 [1.8,3.9]	6.0 [4.6,7.8]	
Other	2.8 [1.9,4.2]	2.4 [1.6,3.6]	6.1 [4.6,8.0]	4.3 [3.1,6.2]	3.9 [2.8,5.5]	3.2 [2.2,4.7]	
*Type of rice consumed (consumers only, n = 1511)*							.46
White	87.5 [82.2,91.4]	87.3 [82.0,91.2]	85.7 [79.4,90.4]	88.2 [82.6,92.2]	89.9 [86.0,92.8]	85.3 [80.5,89.1]	
Brown	12.5 [8.6,17.8]	11.5 [7.7,16.7]	11.7 [7.5,17.7]	10.5 [6.7,15.9]	8.3 [5.7,12.0]	13.2 [9.6,17.8]	
Both	0	1.2 [0.4,3.8]	2.6 [1.0,6.4]	1.3 [0.4,4.3]	1.8 [0.9,3.8]	1.5 [0.7,3.4]	
*Type of pasta consumed (consumers only, n = 1382)*							.48
Regular	91.9 [86.8,95.2]	91.7 [87.0,94.8]	95.3 [91.8,97.4]	96.5 [92.2,98.5]	92.7 [88.0,95.6]	90.7 [86.3,93.8]	
Wholemeal	6.5 [3.7,11.2]	7.3 [4.4,11.9]	3.2 [1.6,6.4]	3.3 [1.3,7.7]	6.4 [3.6,10.9]	7.8 [4.9,12.1]	
Both	1.6 [0.4,5.7]	1 [0.2,3.8]	1.4 [0.5,4.0]	0.2 [0.0,1.5]	1 [0.2,4.0]	1.5 [0.6,3.6]	
*Proportion consuming whole-grain cereals (brown bread, wholemeal bread, multigrain bread, whole-grain bread, brown rice, wholemeal pasta, n = 7044)*							
No whole-grain cereals	51.1 [47.6,54.5]	54.6 [51.3,57.9]	65.6 [62.3,68.6]	53.0 [49.6,56.3]	38.2 [35.0,41.6]	37.2 [44.6,47.5]	<.001
Any whole-grain cereals	42.5 [39.1,45.9]	39.0 [35.8,42.2]	25.6 [22.8,28.5]	34.4 [31.3, 37.6]	49.9 [42.6, 48.9]	45.7 [42.6, 48.9]	
Missing data	6.4 [5.8,8.2]	6.4 [4.9,8.3]	8.9 [7.1,11.0]	12.7 [10.6,15.0]	11.9 [10.0,14.0]	17.1 [14.8,19.6]	
*Amount consumed (among consumers)* ^a^							
Bread (slices), *n* = 5590	4.1 [3.7,4.4]	4.1 [4,4.3]	3.2 [3,3.4]	3.1 [3,3.2]	3.5 [2.1,4.8]	2.4 [2.3,2.5]	<.001
Bread substitute (serve), *n* = 1600	2.4 [2.0,2.7]	2.7 [2.2,3.2]	3.2 [2.7,3.6]	3.6 [2.9,4.3]	3.7 [2.9,4.5]	3.6 [3.0,4.3]	<.001
Rice (cups), *n* = 1768	1.5 [1.3,1.6]	1.4 [1.3,1.5]	1.4 [1.3,1.5]	1.3 [1.2,1.5]	1.1 [1.0,1.2]	1.1 [1.0,1.2]	<.001
Pasta (cups), *n* = 1439	1.5 [1.4,1.7]	1.5 [1.4,1.7]	1.8 [1.6,2.0]	1.4 [1.3,1.6]	1.3 [1.2,1.4]	1.3 [1.2,1.4]	<.001
Cereal (cups), *n* = 3552	1.9 [1.8,2.1]	1.9 [1.8,2]	2.1 [1.9,2.3]	2.0 [1.8,2.2]	1.8 [1.7,2]	1.8 [1.7,1.9]	.07

Results are survey design-based percentage and except ^a^ in which results are mean. ^b^ *P* values were derived from survey design-based Pearson chi square test for comparisons for difference in prevalence, and Bonferroni test for difference in mean. “NA” data were not available. Among bread consumers, the median serve of bread consumed was 2 serves [interquartile range 2–4], with 2 [2, 3] of female, and 3 [2–4] of male. Among bread substitute consumers, the median serve of bread consumed was 2 serves [interquartile range 2–4], and the same with male and female

### Bread

The proportion of respondents consuming bread decreased from 88.4% in 1995 to 70.4% in 2012 (*p* < .001). Among bread consumers, the amount eaten decreased from 4.1 slices in 1995 to 2.4 in 2012 (*p* < .001). Thirty nine percent of respondents consumed whole-grain bread and this did not change over time. Overall, 22% of respondents ate bread substitutes (bread muffins, pita bread, crumpets, scones, or crackers). Among the consumers, the mean servings of bread substitutes rose from 2.4 serves in 1995 to 3.6 serves in 2004 (*p* < .001).

### Rice and pasta

The proportion of respondents eating rice increased from 22.7% in 1995 to 25.7% in 2012 (*p* = .02) and 85–90% choose white rice. Among rice consumers, the amount eaten decreased from 1.5 cups in 1995 to 1.1 cups in 2012 (*p* < .001). Across all surveys, 21% of respondents ate pasta on the day prior to the survey and 7% chose wholemeal varieties. Among pasta consumers, the amount eaten decreased from 1.8 cups in 2001 to 1.3 cups in 2009 and 2012 (*p* < .001).

### Factors associated with cereal food consumption

Multiple logistic regression modelling showed a step-wise decrease in the likelihood of consuming bread over time (Odds Ratio OR = 0.31; 95% Confidence Interval; 0.24–0.40 in 2012 versus 1995) and that those more likely to consume bread were male (OR = 1.44; 1.23–1.68), shown in Table 3. The likelihood of consuming rice decreased with age (OR = 0.60; 0.43–0.84 in those aged 55–64 years compared to 18 to 24 years) and those more likely to consume rice were born outside of Australia, the UK and Ireland (OR = 2.90; 2.37–3.56), University or high school educated (OR = 1.56; 1.27–1.92 and OR = 1.27; 1.00–1.61 respectively). Obese respondents were less likely to consume rice than those of a healthy weight (OR = 0.71; 0.57–0.87) as were those living outside the metropolitan area (OR = 0.83; 0.70–0.99). Pasta consumers were less likely to be born in the UK and Ireland than in Australia (OR = 0.74; 0.60–0.92). Overweight or obese respondents were less likely to eat pasta than those who were a healthy weight (OR = 0.81; 0.68–0.98 and OR = 0.78; 0.63–0.96 respectively), shown in Table 3.

**Table 3:** Factors associated with self-reported cereal food consumption (bread and alternatives, rice, pasta, breakfast cereal, wholegrain versions) on day prior to survey, Western Australian Nutrition Monitoring Survey Series (NMSS)

Consumed on the day prior	Bread and alternatives *(n = 7040)*	Rice *(n = 6629)*	Pasta *(n = 6654)*	Breakfast cereal *(n = 7009)*	Whole-grain versions (*n = 6178)*
	OR [95% CI]	OR [95% CI]	OR [95% CI]	OR [95% CI]	OR [95% CI]
Year of survey					
1995	1.00	1.00	1.00	1.00	1.00
1998	0.86 [0.64,1.16]	0.90 [0.70,1.17]	1.22 [0.95,1.56]	0.77 [0.64,0.94]^a^	0.81 [0.66,0.99]^a^
2001	0.61 [0.46,0.81]^c^	0.90 [0.69,1.17]	1.41 [1.10,1.81]^b^	0.82 [0.67,1.01]	0.44 [0.35,0.54]^c^
2004	0.46 [0.35,0.60]^c^	1.01 [0.85,1.43]	1.20 [0.93,1.55]	0.86 [0.71,1.06]	0.69 [0.56,0.86]^c^
2009	0.42 [0.32,0.54]^c^	1.27 [0.97,1.65]	1.02 [0.79,1.32]	0.79 [0.65,0.97]^a^	1.27 [1.02,1.58]^a^
2012	0.31 [0.24,0.40]^c^	1.17 [0.90,1.52]	0.99 [0.76,1.27]	0.72 [0.58,0.90]^b^	1.14 [0.91,1.41]
Sex					
Female	1.00			1.00	
Male	1.44 [1.23,1.68]^c^			1.18 [1.04,1.34]^a^	
Age (years)					
18–24		1.00		1.00	1.00
25–34		0.95 [0.70,1.30]		1.02 [0.80,1.30]	1.03 [0.79,1.35]
35–44		0.81 [0.61,1.09]		0.97 [0.77,1.23]	1.05 [0.82,1.36]
45–54		0.85 [0.62,1.15]		1.13 [0.89,1.45]	1.32 [1.01,1.72]^a^
55–64		0.60 [0.43,0.84]^c^		1.59 [1.23,2.05]^c^	1.74 [1.33,2.28]^c^
Education					
Less than high school		1.00		1.00	1.00
High school		1.27 [1.00,1.61]^a^		1.30 [1.09,1.55]^b^	1.19 [0.97,1.45]
TAFE/Certificate/Diploma		1.07 [0.83,1.36]		1.35 [1.13,1.62]^c^	1.23 [1.01,1.50]^a^
University Degree		1.56 [1.27,1.92]^c^		1.39 [1.19,1.63]^c^	1.67 [1.41,1.98]^c^
Currently in paid employment					
No				1.00	
Yes				0.84 [0.73,0.97]^a^	
Country of birth					
Australia		1.00	1.00	1.00	
UK/Ireland		1.00 [0.81,1.23]	0.74 [0.60,0.92]^b^	1.10 [0.94,1.29]	
Other countries		2.90 [2.37,3.56]^c^	0.91 [0.73,1.14]	0.77 [0.65,0.92]^b^	
Residential area					
Metropolitan		1.00			
Rest of the state		0.83 [0.70,0.99]^a^			
BMI category					
Normal weight or under		1.00	1.00		
Overweight		1.01 [0.84,1.21]	0.81 [0.68,0.98]^a^		
Obese		0.71 [0.57,0.87]^c^	0.78 [0.63,0.96]^a^		
Level of attention paid to health aspect of diet					
Pay a lot of attention				1.00	1.00
Take a bit of notice				0.79 [0.70,0.90]^c^	0.54 [0.47,0.61]^c^
Don’t think about				0.38 [0.29,0.49]^c^	0.33 [0.25,0.45]^c^

Results are odds ratio (OR) from binary logistic regressions. ^a^ *p* < .05; ^b^ *p* < .01; ^c^ *p* < .001. Full models include the following variables: survey years, age group, sex, education level, household income, employment status, country of birth, residential area, BMI categories, and how they felt about health aspect of their diet. Variables with *p* value < .05 were retained in the final models and reported in the table with the exception of survey year which remained in final models regardless its significant level

Breakfast cereal consumption prevalence decreased in 2009 and 2012 compared to 1995 (OR = 0.79; 0.65–0.97 and OR = 0.72; 0.58–0.90 respectively). Breakfast cereal consumers were more likely to be male (OR = 1.18; 1.04–1.34), aged 55–64 years compared to 18–24 years (OR = 1.59; 1.23–2.05), and to have a high school (OR = 1.30; 1.09–1.55), college (OR = 1.35; 1.13–1.62), or tertiary education (OR = 1.39; 1.19–1.63). Breakfast cereal consumers were less likely to be unemployed (OR = 0.84; 0.73–0.97), born in countries other than Australia/UK/Ireland (OR = 0.77; 0.65–0.92). Respondents who did not think about the health aspects of their diet (OR = 0.38; 0.29–0.49) or only took a bit of notice (OR = 0.79; 0.70–0.90) were less likely to consume breakfast cereal than those who paid a lot attention, shown in Table 3.

Whole-grain consumption prevalence decreased between 1998 and 2004 compared to 1995 (OR = 0.81; 0.66–0.99, OR = 0.44; 0.35–0.54, and OR = 0.69; 0.56–0.86 respectively) and increased in 2009 (OR = 1.27; 1.02–1.58). Whole-grain consumers were more likely to be older (45–54 years OR = 1.32; 1.01–1.72 and 55 to 64 years OR = 1.74; 1.33–2.28 compared to 18–24 years), to have a college or tertiary education (OR = 1.23; 1.01–1.50 and OR = 1.67; 1.41–1.98 respectively), and pay a lot of attention to the health aspect of their diet (compared to those who did not think about a healthful diet (OR = 0.33; 0.25–0.45) or those who only took a bit of notice (OR = 0.54; 0.47–0.61)), shown in Table 3.

### Knowledge of dietary recommendations for cereal foods

The proportion of respondents correctly identifying the number of servings of cereal foods recommended per day decreased over time, from 26% in 1995 to only 6.3% in 2012 (*p* < .001), shown in Table 4.

**Table 4:** Attitudes and knowledge towards consumption of cereal food, Western Australian Nutrition Monitoring Survey Series (NMSS)

	1995 % [CI]	1998 % [CI]	2001 % [CI]	2004 % [CI]	2009 % [CI]	2012 % [CI]	*p* value
*Best describes you towards the amount of cereal foods you ate (n = 7032)*							<.001
Currently trying to increase	17.4 [14.9,20.2]	13.2 [11.1,15.6]	10.7 [8.8,12.9]	8.7 [6.9,10.8]	7.1 [5.7,9.0]	8.8 [7.1,10.9]	
Thinking about trying to increase	7.8 [6.2,9.7]	9.2 [7.5,11.2]	7.6 [5.8,9.8]	4.6 [3.4,6.3]	5.3 [4.1,6.7]	7.6 [6.1,9.5]	
Not thinking about/already eat enough	74.9 [71.8,77.8]	77.6 [74.8,80.3]	81.8 [78.9,84.3]	86.7 [84.2,88.8]	87.6 [85.4,89.5]	83.6 [81.0,85.9]	
*In the past 12 months have you tried to change the amount of cereal food you eat (n = 7037)*							<.001
Tried to increase amount I eat	25.4 [22.5,28.5]	22.3 [19.7,25.2]	19.8 [17.3,22.7]	12.4 [10.3,14.8]	11.9 [9.9,14.4]	11.3 [9.5,13.5]	
Tried to decrease amount I eat	4.3 [3.2,5.9]	4.7 [3.4,6.5]	7.8 [6.2,9.7]	16.3 [14.0,18.8]	14.7 [12.6,17.2]	21.7 [19.1,24.5]	
Not tried to change	70.3 [67.1,73.4]	73 [69.9,75.9]	72.4 [69.3,75.3]	71.4 [68.3,74.3]	73.3 [70.3,76.2]	67.0 [63.9,69.9]	
*What do you think is the recommended minimum number of serves of cereal foods that should be eaten each day? (n = 7038)*							<.001
0 serve	0.3 [0.1,1.0]	0	0	0.1 [0.0,0.8]	0.9 [0.5,1.7]	0.9 [0.4,2.1]	
1 serve	19.8 [17.2,22.7]	17.1 [14.7,19.9]	21.3 [18.7,24.3]	19.0 [16.5,21.8]	26.4 [23.5,29.5]	33.1 [30.2,36.1]	
2 serves	23.7 [20.8,26.9]	25.0 [22.2,28.0]	23.4 [20.7,26.4]	31.4 [28.3,34.6]	26.8 [24.0,29.7]	29.0 [26.2,31.9]	
3 serves	19.8 [17.3,22.6]	20.7 [18.2,23.5]	21.3 [18.6,24.1]	17.7 [15.3,20.5]	17.9 [15.4,20.6]	13.4 [11.4,15.7]	
4 serves ^a^	12.6 [10.5,15.1]	13.7 [11.6,16.1]	11.4 [9.4,13.8]	6.1 [4.6,8.0]	6.3 [4.9,8.1]	4.1 [3.0,5.5]	
5 or more serves	13.4 [11.2,16.0]	15.4 [13.2,18.0]	11.6 [9.5,14.0]	7.0 [5.4,9.0]	4.7 [3.6,6.3]	2.2 [1.6,3.2]	
*Which types of cereal foods have you tried to increase? – Among those who said they had tried to increase.*							
Breakfast cereal (*n* = 1140)	61.2 [55.1,67.1]	58.3 [51.4,64.8]	72.7 [65.9,78.6]	80.1 [72.3,86.1]	63.1 [54.2,71.3]	58.6 [49.6,67.0]	.002
Pasta (*n* = 1143)	54.5 [48.2,60.6]	52.5 [45.7,59.1]	40.6 [33.7,47.9]	32.1 [24.2,41.3]	22.3 [15.6,30.9]	16.5 [10.9,24.2]	<.001
Rice (*n* = 1142)	54.1 [47.8,60.2]	47.6 [41.0,54.4]	44.1 [37.0,51.5]	40.4 [32.0,49.4]	33.1 [25.0,42.3]	29.0 [21.8,37.4]	<.001
Bread (*n* = 1141)	20.3 [15.8,25.7]	31 [25.0,37.6]	26.7 [20.7,33.8]	19.0 [12.8,27.4]	16.8 [10.5,25.9]	15.1 [9.7,22.6]	.01

Results are survey design-based percentage and *p* values were derived from survey design-based Pearson chi square test

^a^The minimum number of serves recommended in the Australian Guide to Healthy Eating was four per day(8)

Logistic regression models showed the proportion correctly identifying the recommended number of cereal foods servings decreased from 2004 (Relative Risk Ratio (RRR) = 0.46; 0.35–0.60 in 2004, RRR = 0.37; 0.28–0.48 in 2009, and RRR = 0.20; 0.15–0.27 in 2012) compared to 1995, Table 5. The odds of correctly identifying the recommended number of cereal food servings were lower among males (RRR = 0.77; 0.65–0.99), people over 35 years of age (35–44 years RRR = 0.76; 0.58–0.91, 45–54 years RRR = 0.66; 0.49–0.87 and RRR = 0.53; 0.39–0.73 respectively) and respondents who did not think about the health aspects of their diet (RRR = 0.49; 0.34–0.71). Respondents who held a university degree were twice as likely to correctly identify cereal recommendations than those who had less than high school education (RRR = 1.98; 1.60–2.44).

**Table 5:** Factors associated with correctly identifying the recommended number of servings of cereal foods per day, Western Australian Nutrition Monitoring Survey Series (NMSS)

	Knowledge of recommended cereal intake (≥4 servings versus less than 4 servings)
	OR [95% CI]
Year of survey	
1995	1.00
1998	1.18 [0.94,1.47]
2001	0.83 [0.66,1.05]
2004	0.46 [0.35,0.60]^c^
2009	0.37 [0.28,0.48]^c^
2012	0.20 [0.15,0.27]^c^
Sex	
Female	1.00
Male	0.77 [0.65,0.91]^b^
Age (years)	
18–24	1.00
25–34	0.81 [0.62,1.07]
35–44	0.76 [0.58,0.99]^a^
45–54	0.66 [0.49,0.87]^b^
55–64	0.53 [0.39,0.73]^c^
Education	
Less than high school	1.00
High school	1.50 [1.19,1.89]^c^
Trade/Certificate/Diploma	1.18 [0.89,1.55]
University degree	1.98 [1.60,2.44]^c^
Level of attention paid to health aspect of diet	
Pay a lot of attention	1.00
Take a bit of notice	0.87 [0.73,1.04]
Don’t think about	0.49 [0.34,0.71]^c^

Results are odds ratio (OR) and 95% confidence interval, derived from a binary logistic regression under survey module. Only variables with *p* < .05 from overall Wald test after regression were retained in the final model and reported

^a^ *p* < .05

^b^ *p* < .01

^c^ *p* < .001

### Barriers and promoters of cereal food consumption

The proportion of respondents not thinking about increasing the amount of cereal foods they ate, increased from 74.9% in 1995 to 83.6% in 2012 (*p* < .001), see Table 4. The main reasons respondents said they would increase their cereal food intake were to improve their general health (30.7%), or ‘weight or diet-related’ (24.0%), shown in Table 6. The main barriers to increasing consumption were ‘not liking cereals or not interested’ (9.8%), ‘no time for breakfast’ (5.4%) or ‘not liking eating in the morning or breakfast’ (5.3%).

**Table 6:** Perceived promoters and barriers towards increasing consumption of cereal food, Western Australian Nutrition Monitoring Survey Series (NMSS)

	1995 % [95% CI]	1998 % [95% CI]	2001 % [95% CI]	2004 % [95% CI]	2009 % [95% CI]	2012 % [95% CI]	Overall % [95% CI]	*P* value
*What influences you to try to eat more cereal foods? (n = 1642)*								
Improve health in general	41.8 [35.3,48.5]	27.8 [21.6,35.1]	32.7 [25.7,40.6]	40.0 [31.0,49.7]	30.5 [24.8,36.7]	26.2 [21.8,31.2]	30.7 [28.0,33.5]	.004
Related to weight/diet	11.2 [7.7,16.2]	6.2 [3.8,9.9]	5.8 [3.2,10.4]	7.2 [3.0,16.3]	41.4 [35.2,47.9]	28.4 [23.9,33.4]	24.0 [21.5,26.7]	<.001
Increase carbs/better balance in my diet	5.6 [3.2,9.6]	21.8 [16.0,29.0]	13.2 [8.9,19.2]	7.7 [3.8,15.0]	3.4 [1.7,6.6]	8.5 [5.8,12.3]	8.6 [7.1,10.4]	<.001
To increase fibre intake	8.1 [5.1,12.5]	23.2 [17.3,30.4]	14.6 [9.9,20.9]	15.8 [10.6,22.8]	3.3 [1.6,6.9]	4.6 [2.7,7.9]	8.2 [6.8,9.9]	<.001
Improve fitness/more energy	7.6 [4.7,12.2]	9.9 [6.4,15.1]	9.4 [5.6,15.3]	17.1 [10.6,26.4]	7.8 [4.7,12.7]	4.4 [2.6,7.4]	7.5 [6.1,9.2]	.004
Medical reasons/doctor/dietitian advice	7.8 [5.0,12.0]	12.4 [8.0,18.7]	5 [2.8,8.8]	3.8 [1.7,8.6]	4.5 [2.7,7.7]	6.6 [4.6,9.4]	6.5 [5.3,7.9]	.02
To reduce bowel problems/health reasons	8.1 [5.1,12.6]	8.4 [4.6,14.8]	5.2 [2.7,9.9]	9.1 [4.8,16.5]	2.1 [1.0,4.2]	6.4 [4.1,9.8]	5.8 [4.5,7.3]	.01
Hungry at work/no breakfast	5.6 [3.1,9.9]	10 [6.4,15.2]	16.2 [11.3,22.7]	18.6 [12.1,27.4]	2.7 [1.2,6.2]	1.2 [0.5,2.6]	5.5 [4.4,6.8]	<.001
Adverts/DOH campaign/cooking on TV	4.7 [2.5,8.6]	8.7 [5.4,13.7]	2.5 [1.0,6.1]	6 [2.3,15.0]	1.2 [0.4,3.1]	0.9 [0.4,2.1]	2.7 [2.0,3.6]	<.001
Other	29.1 [23.5,35.4]	15 [10.9,20.3]	25.6 [19.6,32.6]	10.2 [5.7,17.7]	4.7 [2.7,8.2]	10.8 [7.6,15.1]	13.2 [11.4,15.3]	<.001
Don’t know/no particular reason	2.9 [1.0,8.5]	1.4 [0.4,4.5]	3.4 [1.6,7.3]	3.1 [1.0,9.1]	12.8 [8.6,18.5]	14.9 [11.4,19.3]	9.8 [8.0,12.1]	<.001
*What are the main things that make it difficult for you to eat plenty of cereal foods? (n = 5842)*								
Don’t like cereal/not interested	9.1 [7.4,11.2]	5.8 [4.4,7.7]	4.2 [3.1,5.7]	N/A	13.3 [11.2,15.7]	10.8 [9.1,12.8]	9.8 [8.9,10.8]	<.001
No time for breakfast	6.1 [4.6,8.0]	11.2 [9.1,13.6]	8 [6.2,10.2]	N/A	3.4 [2.3,4.8]	3.6 [2.5,5.3]	5.4 [4.7,6.2]	<.001
Don’t like eating in the morning/breakfast	2.8 [1.8,4.1]	6.5 [4.9,8.5]	6.4 [4.9,8.3]	N/A	5.8 [4.4,7.5]	5 [3.7,6.7]	5.3 [4.6,6.1]	.04
The time to prepare	3.5 [2.4,5.1]	4.2 [3.0,5.8]	3.7 [2.6,5.2]	N/A	6.8 [5.0,9.1]	5.5 [4.1,7.3]	5.2 [4.4,6.1]	.03
Too expensive	2.9 [1.7,5.0]	0.5 [0.2,1.1]	0	N/A	0.8 [0.4,1.5]	1.8 [1.1,2.9]	1.3 [0.9,1.7]	<.001
The effort to prepare	1.3 [0.7,2.3]	0	0	N/A	1.2 [0.7,2.1]	1.9 [1.1,3.4]	1.2 [0.8,1.7]	.005
Other	10.9 [9.0,13.1]	4.3 [3.2,5.8]	0	N/A	3.3 [2.3,4.9]	8.7 [7.0,10.8]	5.8 [5.1,6.7]	<.001
Don’t know	1.5 [0.9,2.6]	1.8 [1.1,3.1]	4.5 [3.2,6.3]	N/A	2.3 [1.5,3.5]	1.2 [0.8,2.0]	2.1 [1.7,2.5]	<.001
Nothing/already eat enough	63.6 [60.1,66.9]	69.2 [66.0,72.3]	75.0 [71.8,77.9]	N/A	62.9 [59.6,66.1]	65.8 [62.7,68.8]	66.3 [64.8,67.9]	<.001

Results are survey design-based percentage and *p* values were derived from survey design-based Pearson chi square test

The proportion who had tried to decrease the amount of cereal foods they ate increased from 4.3% in 1995 to 21.7% in 2012 (*p* < .001). Most respondents had not tried to change their cereal intake in the past year and of those who had tried to increase cereals, most had tried to increase breakfast cereal intake (58.6% to 80.1%). The proportion who tried to increase the amount of pasta they ate in 2012 was less than in 1995 (16.5% compared to 54.5%, (*p* < .001)).

### Factors associated with attempts to change cereal foods intake

Multinomial logistic regressions showed that respondents were 11 times more likely in 2012 to have tried to decrease their cereal food intake compared to 1995 (RRR = 10.88; 6.81–17.4), see Table 7. Overweight and obese respondents were more likely to have tried to increased their cereal intake in the last 12 months compared to those of a healthy weight (RRR = 1.65; 1.22–2.24 and RRR = 1.88; 1.35–2.63 respectively) as were those who identify the correct number of cereal serves ((RRR = 1.56; 1.09–2.23). Males (RRR = 0.46; 0.35–0.60), older respondents (aged 55–64 years and 45–54 years, RRR = 0.55; 0.33–0.93 and RRR = 0.61; 0.38–1.00 compared to those 18–25 years respectively) were likely to have tried to decreased their cereal intake in the last 12 month. Respondents who did not pay attention to the health aspects of the food they eat and those that only took a bit of notice were more likely to have tried to increase their cereal food intake in the previous 12 months (RRR = 0.43; 0.22–0.84 and RRR = 0.55; 0.42–0.71 respectively) but also less likely to be currently trying to eat more cereal foods (RRR = 0.51; 0.32–0.81).

**Table 7:** Factors associated with perception of current cereal foods consumption and attempts to change intake, Western Australian Nutrition Monitoring Survey Series (NMSS)

	Best describes current cereal consumption (*n* = 7030)		Tried to change the amount of cereal foods eaten in past 12 months (*n* = 6684)	
	Currently trying to eat more RRR [95% CI]	Thinking about trying to eat more RRR [95% CI]	Tried to decrease RRR [95% CI]	Tried to increase RRR[95% CI]
	Versus “not thinking about/already eat enough/thinking to decrease”		Versus “have not tried to change”	
Year of survey (1995, ref., RRR = 1.00)				
1998	0.74 [0.56,0.97]^a^	1.13 [0.80,1.58]	1.54 [0.90,2.63]	1.14 [0.89,1.46]
2001	0.56 [0.42,0.75]^c^	0.88 [0.60,1.28]	2.57 [1.58,4.20]^c^	1.25 [0.97,1.62]
2004	0.44 [0.33,0.60]^c^	0.52 [0.34,0.78]^b^	9.19 [5.74,14.71]^c^	1.97 [1.47,2.63]^c^
2009	0.37 [0.27,0.50]^c^	0.61 [0.42,0.89]^b^	6.62 [4.06,10.80]^c^	1.96 [1.45,2.65]^c^
2012	0.50 [0.37,0.69]^c^	0.92 [0.64,1.35]	10.88 [6.81,17.4]^c^	1.84 [1.38,2.46]^c^
Sex (Female, ref., RRR =1.00)				
Male			0.46 [0.35,0.60]^c^	1.04 [0.87,1.24]
Age (years) (18–24, ref., RRR =1.00)				
25–34	0.95 [0.66,1.37]	0.87 [0.56,1.34]	0.72 [0.44,1.17]	1.20 [0.87,1.66]
35–44	0.95 [0.66,1.36]	0.95 [0.63,1.44]	0.67 [0.42,1.06]	1.37 [1.00,1.88]^a^
45–54	0.81 [0.55,1.19]	0.98 [0.64,1.49]	0.61 [0.38,1.00]^a^	1.39 [1.00,1.94]
55–64	0.50 [0.32,0.77]^b^	0.74 [0.47,1.17]	0.55 [0.33,0.93]^a^	1.77 [1.23,2.55]^b^
Body mass index (normal weight or under, ref., RRR =1.00)				
Overweight			1.65 [1.22,2.24]^c^	0.93 [0.76,1.14]
Obese			1.88 [1.35,2.63]^c^	0.70 [0.55,0.88]^b^
Attention paid to the health aspect of diet (Pay a lot of attention, ref., RRR =1.00)				
Take a bit of notice	1.12 [0.91,1.39]	1.61 [1.25,2.06]^c^	0.55 [0.42,0.71]^c^	0.90 [0.75,1.09]
Don’t think about	0.51 [0.32,0.81]^c^	1.55 [1.02,2.37]^a^	0.43 [0.22,0.84]^a^	1.86 [1.25,2.77]^b^
Perceived recommended serves of cereal (0–4 serves, ref., RRR =1.00)				
≥ 4 serves			1.56 [1.09,2.23]^a^	1.15 [0.93,1.43]
Don’t know			1.84 [1.12,3.05]^a^	1.70 [1.20,2.41]^b^

Results are relative risk ratios (RRR) and 95% confidence interval regarding self-perceived current behaviour towards cereal intake and their behaviour in the past 12 months towards cereal foods consumption. Results are from two multinomial logistic regressions using the Stata survey module. Full models include the following variables: survey years, age group, sex, education level, household income, employment status, country of birth, residential area, whether living with children, BMI categories, and their perceived recommended serves per day for cereal foods. Variables with *p* value < .05 were remained in the final models and reported in the table with the exception of survey year which were remained in final models regardless its significant level

## Discussion

The proportion of Western Australian adults consuming cereal foods has decreased over the past two decades. The amount eaten has also decreased, yet most adults were unaware of the recommended intake and believed they were eating enough. In 2012, only 6% were able to identify the correct number of servings for cereals.

Western Australians were more likely to eat bread than any other type of cereal food although the proportion of adults eating bread declined from 88.4% in 1995 to 70.4% in 2012. A similar decline in bread consumption is evident from Australian dietary surveys using 24-h dietary recall methods, from 81% of adults consuming bread in 1995 to 65% in 2011–12 [10, 11]. The amount of bread consumed also decreased from 4.1 slices in 1995 to 2.6 slices in 2012 in the current study, compared to a decline in the national surveys from 2.7 slices (109 g) of regular bread and rolls in 1995 to 2.2 slices (88 g) in 2011/12 [10, 11]. While the proportion consuming bread alternatives (e.g. bread muffins, pita bread, or rice crackers) in the current study remained unchanged, the amount of these types of bread alternatives consumed increased over the two decades.

Cultural similarities and differences in bread consumption are seen when the current study findings are compared to intakes in other countries. Adult bread consumption in the UK declined in the decade preceding 2011 by around 12% [30] to an average of 186 g / day [31], whereas in 2011 98% of the Swedish population ate bread and intake had not changed since 1989 [32]. Despite this, in 1992–1998 the average Swedish bread consumption of 56 g / day was less than the intake from surrounding countries Norway and Denmark (127 g / day and 142 g / day respectively) [33]. Wheat bread intake in the United States (US) in 2003–6 NHANES accounted for 7.2% of energy intake [18], similar to Australian dietary surveys.

The current study found the proportion consuming breakfast cereals remained constant at about 50% with no change in the amount consumed (around two cups among those consuming) over the survey years. Australian dietary surveys also show breakfast cereal consumption has remained constant [10, 11], however, in contrast to the current study, they report increasing prevalence of ready-to-eat cereals (from 26.4% in 1995 to 34.5% in 2012) [10, 11]. The difference in prevalence may be due to different survey methodology including the categorisation of breakfast cereals or seasonality differences. In general, UK and Scandinavian national surveys show a lower mean intake of breakfast cereals compared with Australia, an average of 26 g of breakfast cereals per day among UK adults in 2008–11 [31], and Norway, Sweden and Denmark averaged 21 g per day, while Swedish adults consumed 51 g per day [33].

The findings on cereal food consumption are policy relevant, as the Australian Government uses food consumption information to inform food fortification and regulation policy to address public health nutrition deficiencies and prevent neural-tube defects. This has occurred through mandatory fortification of bread flour with thiamin since the 1970s, and folic acid and iodised salt since 2009 [34]. The current study findings suggest that further research is required to explore the impact of declining bread intake on the effectiveness of these regulatory policies.

The proportion of WA adults consuming whole-grain cereal foods increased in 2009 with health conscious eaters and those with higher educational attainment more likely to eat whole-grain foods. The diet of regular whole-grain consumer is likely to be more nutrient dense diet compared with non-consumers [16, 18]. Given the importance of dietary whole-grains to reducing the population risk of non-communicable diseases [5], it is concerning that there has been little change in intake over twenty years. Although a larger proportion of bread consumers selected whole-grain or wholemeal breads, the decline in prevalence and amount of bread eaten suggest there has been no change in the overall population intake of whole-grain bread. This finding is consistent with Australian manufacturing industry reports of a shift toward whole-grain and seeded varieties of bread [35], despite the ongoing popularity of traditional white bread [36]. Bread and breakfast cereals are the main contributors of whole-grains in the current study and in the US and UK [37]. In the UK bread provides almost half and breakfast cereals about one-third of dietary whole-grains [38]. Population surveys reveal cultural differences in whole-grain food consumption which is typically higher in Scandinavian countries than in many European countries, the US [33] and Australia. Only 7% of adults from Denmark, Sweden and Norway [15] did not consume any foods containing whole-grains compared to 46% in this study, 18% of UK adults [16], 42% of US adults [39] and 68% of French adults [22].

Knowledge of the amount of cereal food recommended for good health was low, only 6% of WA adults knew the recommendation in 2012, with most giving an incorrect amount of less than four servings. Consumer misperceptions, typically perpetuated by the media and celebrity diets, that carbohydrates and cereal foods are fattening [30] or bloating or gastrointestinal discomfort indicative of allergy or food intolerances [30, 40] may contribute to declining intakes. About one in ten Australian adults avoided wheat containing foods due to perceived gastrointestinal discomfort without any formal diagnosis in 2011 [40]. Although coeliac disease affects approximately 1 % of the Australian [40], US [41], and European populations [42] and about 13% of Australian [40] and US [43] adults report self-diagnosed gluten sensitivity, there has been a rapid proliferation of wheat-free and gluten-free food products on supermarket shelves in the last decade or so [44]. Some consumers mistakenly believe that gluten free eating assists with weight loss [41]. Combined with low awareness of health benefits of eating whole-grains, these food avoidance perceptions may be contributing to the declining cereal food intake [24]. Further research is needed to understand the impact of popular or fad food and diet trends on perceptions of and adherence to ADG.

Although dietary cereal intake is less than recommended, in 2012, most WA adults were not thinking about increasing the amount they ate and many believed they already ate enough. The low awareness of the recommended cereal intake and misperceptions of dietary adequacy are barriers to adhering to dietary guidelines.

Cost has also been identified as a barrier to consuming whole-grain foods [24] and whole-grain food consumption is lower among Australian low income households [45]. However, whole-grain cereal foods, for example, rice, pasta and bread are priced equivalent to their refined versions, suggesting an opportunity for practical education to encourage consumption. Other barriers to increasing whole-grain consumption include taste [24, 30], particularly for bread [46], lack of understanding of the health benefits, inability to identify whole-grain foods, poor preparation skills, and the influence of family members, particularly young children [24].

All of these barriers could be addressed through a public education program such as the Danish health promotion campaign to increase whole-grain consumption which increased the average whole-grain intake from 36 g to 63 g per day per 10 MJ between 2008 and 2014 [47]. The multi-strategy Danish campaign included: increasing whole-grain content of commercial food products; mass communication to improve consumers’ knowledge about whole-grain foods and their health benefits; and a certification logo on foods with a high whole-grain content (which also met strict criteria for total sugar, fat, and sodium) [47]. Similarly, following the widespread media attention regarding the 2005 US Dietary Guidelines which included a recommended amount of whole-grains for the first time, sales of whole-grain pasta, bread, rice and flour increased and new and reformulated products increased the availability and sales of all whole-grain foods [48]. Food industry action to improve the availability, accessibility, and affordability of whole-grain cereal foods can work in tandem with public education to increase awareness of the need to eat more and the benefits of eating more cereal foods.

ADG promotion has been very limited in Australia with the exception of the government-led comprehensive multi-strategy Gofor2&5© fruit and vegetable social marketing campaign. Triggered by similar consumer misperceptions regarding adequacy of consumption, Gofor2&5© successfully increased awareness of the need to eat more fruit and vegetables with population increases in consumption [49]. Current findings suggest that a similar strategy for cereal foods, particularly for wholegrains, is warranted. The newly formed Australian Government’s *Healthy Food Partnership* has the potential to put such a strategy in place as it brings together the public health sector, food industry and government to improve the dietary habits of Australians [50].

A potential limitation of the current study is the cross-sectional self-report dietary intake methodology which restricts assessment of percentage contribution to total energy intake and the potential for social desirability bias. However, the similarity between the current study findings and the 24 food recall national dietary survey results suggest otherwise. A strength is that surveys have been repeated over time so the population prevalence of consumption, mean intake and consumer beliefs and attitudes have been monitored, particularly in the absence of a routine Australian dietary monitoring system. The cultural difference in dietary intakes described previously, suggest that the study findings are likely to be generalizable only in the Australian context, however, the attitudes and barriers may be similar in other countries.

## Conclusion

Cereal food consumption by Western Australian adults continues to decrease and whole-grain consumption has not improved, yet there is a common misconception of dietary adequacy and low awareness of the amount that should be eaten for good health. The decline in bread consumption is particularly concerning as bread is the primary vehicle for folate, thiamine and iodine fortification. The WA population would benefit from a health promotion intervention to increase awareness of the importance of cereal foods, in particular breads and whole-grains, and assistance with overcoming practical barriers to incorporating these foods into their diet.

## Additional file

Additional file 1: Nutrition Monitoring Survey Series (NMSS) 2012 (PDF 312 kb).

## Abbreviations

ADG: Australian dietary guidelines
BMI: Body mass index
CI: 95% Confidence interval
NMSS: Nutrition Monitor Survey Series
OR: Odds ratio
RRR: Relative Risk Ratio
UK: United Kingdom
US: United States of America
WA: Western Australia/n
