# Supplementary material for: Consumer attitudes and misperceptions associated with trends in self-reported cereal foods consumption: cross-sectional study of Western Australian adults, 1995 to 2012
Source: BMC Public Health. 2017 Jun 26;17:597. doi: 10.1186/s12889-017-4511-5 (PMC5485615; doi:10.1186/s12889-017-4511-5)
Supplement: Additional file 1: — Nutrition Monitoring Survey Series (NMSS) 2012 (PDF 312 kb). [file 12889_2017_4511_MOESM1_ESM.pdf]

Government of Western Australia
Department of Health

Nutrition Monitoring Survey Series (NMSS)
2012 Questionnaire

Department of Health, WA

Nutrition Monitoring Survey Series 2012 Questionnaire

Prepared by the Food Law, Policy and Communications Research Translation Project, with funding from the Chronic Disease Prevention Directorate, Department of Health. This is part of the documentation of the Western Australian Nutrition Monitoring Survey Series (NMSS) 1995 to 2012, Grant Agreement G06095.

© Department of Health in Western Australia, 2013.

For more information about this questionnaire or requesting information from the Nutrition Monitoring Survey Series please contact:

Dr. Christina Pollard
Curtin University
Phone: (08) 9266 1142
Email: C.Pollard@curtin.edu.au

Nutrition Monitoring Survey 2012

There are 4 parts to this questionnaire.
1. Perceptions relating to diet
2. Consumption day prior
3. Support for Government Nutrition Policies/Activities
4. Demographics

Hello. I'm _____ calling on behalf of the Department of Health regarding the study we are conducting in Western Australia on important nutrition issues.

I need to speak to the person in the household aged between 18 and 64 years who was the last to have a birthday. Would that be you or somebody else? Interviewer note: If it is someone else, repeat the introduction.

Interviewer note: If there is no one in the age range, this call is to be designated out or range on the response sheet.

[Read To All Respondents]

I can assure you that information given will remain confidential. The answers from all people interviewed will be gathered together and no individual answers will be published or passed on. On average the survey takes 20 minutes. Participation in the survey is voluntary. You may withdraw from the survey at any time and may refuse to answer any questions as you wish.

Case ID __________

Station # __________

Day of week

1. Sunday
2. Monday
3. Tuesday
4. Wednesday
5. Thursday
6. Friday
7. Saturday

Project: Nutrition

**Q1. We recently sent you a letter telling you about the survey.
Did you receive the letter we sent you? (Single Response)
[IF NO, give basic explanation]**

0. No
1. Yes
998. Don't know
999. Refused

BASIC EXPLANATION

The letter invited you to take part in an important study on nutrition being conducted by the Department of Health. It was sent to your household to let you know that we would be contacting you by phone.

Your responses will form part of a picture of nutrition in your local community. The results of the survey will be used to help us keep track of nutrition of Western Australians. That way we can better plan and develop services to meet local needs. On average the survey takes 20 minutes, and participation is voluntary.

DEM1 Is the respondent...? (Single Response)

0. Female
1. Male

DEM2 What was your age last birthday? (Single Response. Refused= 999)

Enter age_____ (years)

Interviewer note: If asked why this is necessary, explain that the results need to be reflective of the WA community and age and sex are used to weight the information so that a 'true picture' is formed about nutrition in WA.

Part 1- Perceptions relating to diet

Now we are going to ask you a few questions about your opinions about nutrition.

opin In your opinion, what do you think are the major problems with the average Australian diet? Try to think of all the problems. (Single Response. Do not read- may probe with ANYTHING ELSE, after initial response.)

1. Too much alcohol/beer
2. Too much fast/junk food
3. Too much fat/wrong sorts of fat/saturated fats
4. Too much salt
5. Too much sugar/sweet things/chocolates/confectionery/cakes/lollies
6. Not enough fruit/vegies/salads
7. Not eating a balanced diet/not eating wide enough variety of foods
8. No time to eat-cook properly/too lazy to cook/rely too much on pre-prepared foods
9. Buying healthy food is too expensive

10.Food safety

11. Nothing/none/no problems

13. Other

Other specified ________________________________

998. I don't know if there are any problems

999. Refused

Now for a few questions about you and how you would describe yourself. These questions will help us to interpret the results about nutrition.

opin15 Which of the following statements would best describe how you feel about your diet? (Read options. Single Response)

1. I pay a lot of attention to the health aspect of the food I eat to make sure my diet is as healthy as possible
2. I take a bit of notice of the health aspect of the food I eat to make sure I have a fairly good diet
3. I don't really think much about the health aspect of food I eat

998. Can't remember/Don't know
999. Refused

opin16 Which of the following best currently describes you? (Read options. Single Response)

1. I am currently trying to eat more fruit
2. I am thinking about trying to eat more fruit
3. I am not thinking about increasing the amount of fruit I eat
4. I already eat enough fruit

998. Can't remember/Don't know
999. Refused

opin17 Which of the following best currently describes you? (Read options. Single Response)

1. I am currently trying to eat more vegetables
2. I am thinking about trying to eat more vegetables
3. I am not thinking about increasing the amount of vegetables I eat
4. I already eat enough vegetables

998. Can't remember/Don't know
999. Refused

opin18 Which of the following best currently describes you? (Read options. Single Response)

1. I am currently trying to eat more cereal foods
2. I am thinking about trying to eat more cereal foods
3. I am not thinking about increasing the amount of more cereal foods I eat
4. I already eat enough cereal foods

998. Can't remember/Don't know
999. Refused

opin19 Which of the following best currently describes you? (Read options. Single Response)

1. I am currently trying to eat less fat and fatty foods
2. I am thinking about cutting down on the amount of fat and fatty foods I eat
3. I am not thinking about cutting down on the amount of fat and fatty foods I eat
4. I already eat a low fat diet

998. Can't remember/Don't know
999. Refused

opin20 Which of the following statements best describes up? (Read options. Single Response)

1. I am currently trying to increase the amount of calcium rich foods I eat
2. I am thinking about trying to increase the amount of calcium rich foods I eat
3. I am not thinking about increasing the amount of calcium rich foods I eat
4. I already eat enough calcium rich food

998. Can't remember/Don't know
999. Refused

opin21 Which of the following best currently describes you? (Read options. Single Response)

1. I am currently trying to lose weight
2. I am currently trying to gain weight
3. I am thinking about trying to lose weight
4. I am thinking about trying to gain weight
5. I am not thinking about trying to lose or gain weight

998. Can't remember/Don't know
999. Refused

food6 Which of the following best describes your cooking skills? (Read options. Single Response)

1. Can't cook
2. Can boil an egg, or BBQ meat or heat frozen meals
3. Can cook basic meat and 3 veg type meals
4. Can cook a wide variety of meals
5. Can cook almost anything

998. Unsure/Don't Know/Can't Remember
999. Refused

Part 2- Consumption day prior

Now I would like to ask you some questions what you ate yesterday.

con1 How many pieces of fruit did you eat yesterday? A piece of fruit would be, for example, an apple, a small bunch of grapes, 3 prunes, a quarter of a rock melon or half a cup of stewed, pureed or canned fruit. (Single Response. Code NONE as 0 and less than one serve a day as 991. Code Unsure/Don't Know/Can't remember as 998 and Refused as 999)

Enter number of pieces ___

con2 How many different types of vegetables, did you eat yesterday? Please remember to include salad, fresh, frozen, canned, raw and cooked vegetables. (Single Response. Record no vegetables as 0, don't remember as 998 and refused as 999)

______

con3 How many serves of vegetables do you usually eat each day? A serve of vegetables is equal to half a cup of cooked vegetables or 1 cup of salad.
(Single Response. Code NONE as 0 and less than one serve a day as 991. Code Unsure/Don't Know/Can't remember as 998 and Refused as 999)

Enter number of serves _____

cer1 Did you eat any bread yesterday, don't include rolls? (Single Response)

0. No **(Go to cer2)**
1. Yes
998. Can't remember **(Go to cer2)**
999. Refused **(Go to cer2)**

cer1a How many slices of bread did you eat? (Single Response. Record don't know as 998 and refused as 999)

______

cer2 Did you eat any bread rolls yesterday? (Single Response)

0. No
1. Yes
998. Can't remember **(Go to cer5)**
999. Refused **(Go to cer5)**

cer4 Were they large rolls or small rolls? (Single Response)

1. Large rolls
2. Small rolls
3. Both
998. Can't remember
999. Refused

cer4a How many rolls did you eat yesterday? (Single Response. Record can't remember as 998 and refused as 999)

______

cer5 What type of bread or bread rolls did you mainly eat yesterday? Was it brown, wholemeal, multigrain, wholegrain, white, white with extra fibre, or another type? (Read options. Single Response)

1. Brown or wholemeal
2. Multigrain or wholegrain
3. White with extra fibre
4. White
5. Other
998. Can't remember
999. Refused

cer5o What was the other type of bread or bread roll did you mainly eat yesterday?

____

cer6 Did you eat any bread substitute such as rice cakes, crumpets, scones, crackers, damper, flat breads like pita bread, lavash bread or bread wraps yesterday? (Single Response.)

0. No **(Go to cer16)**
1. Yes
998. Can't remember **(Go to cer16)**
999. Refused **(Go to cer16)**

I will read out a list of bread substitutes and I would like you to tell me if you ate any of these yesterday and how many. (Interviewer note: For all amounts, round any part portions up, so 1.5=2)

cer7 Did you eat any bread muffins? (Single Response)

0. No **(go to cer8)**
1. Yes
998. Can't remember **(go to cer8)**
999. Refused **(go to cer8)**

cer7a How many muffins did you eat? (Single Response. Record don't know as 998 and refused as 999)

____

cer8 Did you eat any flat bread like pita bread, lavash bread or bread wraps? (Single Response)

0. No **(go cer9)**
1. Yes
998. Can't remember **(go cer9)**
999. Refused **(go cer9)**

cer8a How many pieces of flat bread did you eat? (Single Response. Record don't know as 998 and refused as 999)

____

cer9 Did you eat any crumpets? (Single Response)

0. No **(go to cer10)**
1. Yes
998. Can't remember **(go to cer10)**
999. Refused **(go to cer10)**

cer9a How many crumpets did you eat? (Single Response. Record don't know as 998 and refused as 999)

____

cer10 Did you eat any scones? (Single Response)

0. No **(go to cer11)**
1. Yes
998. Can't remember **(go to cer11)**
999. Refused **(go to cer11)**

cer10a How many scones did you eat? (Single Response. Record don't know as 998 and refused as 999)

______

cer11 Did you eat any rice cakes? (Single Response)

0. No **(go to cer12)**
1. Yes
998. Can't remember **(go to cer12)**
999. Refused **(go to cer12)**

cer11a How many rice cakes did you eat? (Single Response, Record don't know as 998 and refus as 999)

______

cer12 you eat any large crackers? (Single Response. Interview note: a large cracker is one the size of a SAO cracker or larger...)

0. No **(go to cer13)**
1. Yes
998. Can't remember **(go to cer13)**
999. Refused **(go to cer13)**

cer12a How many large crackers did you eat? (Single Response. Record don't know as 998 and refused as 999)

______

cer13 Did you eat any small crackers? (Single Response. Interview note: a small cracker is one the size of a Ritz cracker or a Sakata cracker or a saltine...)

0. No **(go to cer14)**
1. Yes
998. Can't remember **(go to cer14)**
999. Refused **(go to cer14)**

cer13a How many small crackers did you eat? (Single Response. Record don't know as 998 and refused as 999)

______

cer14 Did you eat any damper? (Single Response)

0. No **(go to cer15)**
1. Yes
998. Can't remember **(go to cer15)**
999. Refused **(go to cer15)**

cer14a How many slices of damper did you eat? (Single Response. Record don't know as 998 and refused as 999)

______

cer15 Did you eat any other bread substitute? (Single Response)

0. No **(go to cer16)**
1. Yes
998. Can't remember **(go to cer16)**
999. Refused **(go to cer16)**

cer15a What was it?

____

cer15b How much of it did you eat? (Single Response. Record don't know as 998 and refused as 999)

______

cer16 How many cups of cooked rice did you eat yesterday? (Single Response. Record none as 0, don't know as 998 and refused as 999 go to cer17)

______

cer16a What type of rice did you eat? (Read options. Single Response)

1. White
2. Brown
3. Both
998. Can't remember
999. Refused

cer17 How many cups of cooked pasta or spaghetti did you eat yesterday? (Single Response. Record non as 0, don't know as 998 and refused as 999 and go to cer18)

______

cer17a What type of pasta or spaghetti was it? (Read options. Single Response)

1. Regular (not including wholemeal)
2. Wholemeal
3. Both
998. Can't remember
999. Refused

I would like to ask you about the kinds of breakfast cereal you ate yesterday.

cer18 How many different types of cereal did you eat yesterday? (Single Response. Record none as 0, don't know as 998 and refused as 999 and go to dairy 1)

______

cm1 What type of breakfast cereal did you eat? (Single Response. Interviewer code according to the code list provided)

1. Oats and porridge
2. All bran or other fibre based without fruit
3. Fruit based with any combination
4. Mixed grain such as Antioxidine Guardian Healthwise
5. Muesli or granola
6. Cornflakes, Special K, Wheaties or wheat flakes
7. Sweet cereal, e.g. Honey oats, Coco pops
8. Cheerios, Rice puffs rice based
9. Wheat Bix, Vita Brits, other wheat biscuits
10. Other such as gluten free

For porridge or flakes ask cups. For weet-bix, vitabrits etc, ask how many biscuits

cm1a How many cups of ______ did you eat? (Single Response. Record don't know as 998 and refused as 999)

______ **(Go to dairy1)**

cm2 What type of breakfast cereal did you eat? (Single Response. Interviewer code according to the code list provided)

1. Oats and porridge
2. All bran or other fibre based without fruit
3. Fruit based with any combination
4. Mixed grain such as Antioxidine Guardian Healthwise
5. Muesli or granola
6. Cornflakes, Special K, Wheaties or wheat flakes
7. Sweet cereal, e.g. Honey oats, Coco pops
8. Cheerios, Rice puffs rice based
9. Wheat Bix, Vita Brits, other wheat biscuits
10. Other such as gluten free

cm2a How many cups of _____ did you eat? (Single Response. Record don't know as 998 and refused as 999)

______ **(Go to dairy1)**

cm3 What type of breakfast cereal did you eat? (Single Response. Interviewer code according to the code list provided)

1. Oats and porridge
2. All bran or other fibre based without fruit
3. Fruit based with any combination
4. Mixed grain such as Antioxidine Guardian Healthwise
5. Muesli or granola
6. Cornflakes, Special K, Wheaties or wheat flakes
7. Sweet cereal, e.g. Honey oats, Coco pops
8. Cheerios, Rice puffs rice based
9. Wheat Bix, Vita Brits, other wheat biscuits
10. Other such as gluten free

cm3a How many cups of _____ did you eat? (Single Response. Record don't know as 998 and refused as 999)

______ **(Go to dairy1)**

This section is about dairy and dairy related products including soy based.

dairy1 How many cups of any type of milk did you have or use yesterday? Include milk used in cereal, tea, coffee, other drinks or cooking, including powdered milk. (Record I didn't use any milk as 0 and fractions of a cup as 1/4=.25; 1/3=.33 1/2=.5 2/3=.66 3/4=.75). If the respondent says that they only used milk in tea or coffee record that a .1, don't know as 998 and refused as 999)

______ **(If 0, go to dairy5)**

dairy2 What was the main type of milk that you used yesterday - this includes milk in tea or coffee? (Read out, single response)

1. Cows milk **(Go to dairy3)**
2. Soy milk **(Go to dairy3)**
3. Other
998. Don't Know/Can't remember **(Go to dairy3)**
999. Refused **(Go to dairy3)**

dairy2a What was the other kind of milk that you used?

____

dairy3 Was the milk (Read options. Single Response)

1. Skim/non fat
3. Low fat/2% fat
5. Whole milk or full fat
998. Don't Know/Can't remember
999. Refused

dairy4 Was the milk mainly? (Read options. Single Response)

1. Flavoured
2. Plain
3. Both
998. Don't Know/Can't remember
999. Refused

dairy5 How many cups of yoghurt did you have yesterday? (Single Response. Record I didn't use any milk as 0 and don't know as 998 and refused as 999 and go to ch1, code fractions of a cup as 1/4=.25; 1/3=.33 1/2=.5 2/3=.66 3/4=.75).

______

dairy6 What type of yoghurt did you eat yesterday? (Read options. Code didn't any yoghurt as 0 and code yukult as 4)

0. Didn't eat any yoghurt yesterday **(Go ch1)**
1. Cow's milk yoghurt **(Go to dairy7)**
2. Sheep's milk yoghurt **(Go to dairy7)**
3. Soy milk yoghurt **(Go to dairy7)**
4. Other type
998. Don't Know/Can't remember **(Go to dairy7)**
999. Refused **(Go to dairy7)**

dairy6a Specify other here

____

dairy7 What was the yoghurt ? (Read options. Single Response)

1. Skim/non fat
3. Low fat/2% fat
5. Whole milk or full fat
998. Don't Know/Can't remember **(Don’t read)**
999. Refused **(Don’t read)**

dairy8 Was the yoghurt mainly? (Read options. Single Response)

1. Flavoured
2. Plain
3. Both
998. Don't Know/Can't remember **(Don't read)**
999. Refused **(Don't read)**

I would like to ask you about the kinds of cheese you ate yesterday.

ch1 How much soft cheese such as ricotta, cottage, brie, camembert, castello, cream cheese, did you eat yesterday? A serve of soft cheese is equal to one rounded tablespoon. (Single Response. Record none as 0, don't know as 998 and refused as 999)

______

Interviewer note: If the person says that they didn't eat any cheese at all yesterday, code 0 in ch1, ch2 and ch3.

ch2 How much hard cheese such as cheddar, edam did you eat yesterday? A serve of hard cheese is equal to one slice or a 2.5 cm cube. (Record none as 0, don't know as 998 and refused as 999)

______

ch3 How much grated cheese yesterday such as grated cheddar, mozzarella, parmesan or Romano did you eat yesterday? A serve of grated cheese is equal to a rounded tablespoon. (Record none as 0, don't know as 998 and refused as 999)

______

ch4 Was the cheese you ate yesterday mainly regular, reduced fat or low fat cheese? (Read options. Single Response)

1. Low fat, like ricotta or cottage
2. Reduced fat cheese
3. Regular cheese
998 Don't Know/Can't remember **(Do not read)**
999 Refused **(Do not read)**

I am going to ask you about the red meat that you ate yesterday. I am only interested in whether or not you ate any beef, lamb or veal.

mt1 Did you eat any beef, lamb or veal yesterday? (Single Response)

0. No **(Go to Fish 1)**
1. Yes

mt2 How much beef, lamb or veal as a piece of steak did you eat yesterday? A steak serve is a piece of steak that is about the size on your palm (Single Response. Record none as 0, don't know as 998 and refused as 999).

Serves steak ________

mt3 How many beef, lamb or veal chops did you eat yesterday? 1 serve is 2 small chops (Single Response. Record none as 0, don’t know as 998 and refused as 999.

Serves chops ________

mt4 How much roast beef, lamb or veal did you have yesterday? 1 serve is 3 slices of roast meat. (Single Response. Record none as 0, don’t know as 998 and refused as 999).

Serves roast ________

mt5 How many beef, lamb or veal, hamburger/patties did you eat yesterday? 1 serve is 1 ½ patties (Single Response. Record none as 0, don’t know as 998 and refused as 999).

Serves patties ________

mt6 How much beef, lamb or veal, mince did you eat yesterday? 1 serve is ½ cup of mince (Single Response. Record none as 0, don’t know as 998 and refused as 999).

Serves mince ________

mt7 How many beef, lamb or veal, sausages did you eat yesterday? 1 serve is 1 thin sausages (1 thick is 2 serves) (Single Response. Record none as 0, don’t know as 998 and refused as 999).

Serves sausages ________

Now I will ask you about the fish you ate yesterday. I am only interested in fish not in all seafood, that is I don’t need to know if you ate prawn or oysters or any other crustaceans.

fish1 Thinking only of fish fillets or tinned fish such as tuna or salmon, how much fish did you eat yesterday? 1 serve of fish weighs 115 grams or 1 100 gram tin (Single Response. Record none as 0, don’t know as 998 and refused as 999).

Serves fish ________

oth1 How many sweet biscuits did you eat yesterday? (Single Response. Record didn't eat any biscuits as 0, don't know as 998 and refused as 999)

____

oth2 In total, how many cans, bottles, glasses or cups of soft drink, energy drinks, sports drinks, flavoured mineral water or vitamin water, did you drink yesterday? Please say how large the container was in your answer, for example, 2 375 ml cans of RedBull or 1 litre bottle of Coke. **(Record didn't drink any as 0 in the first field (cans), don't know as 998 and refused as 999) (Interviewer - prompt for size of can or bottle)**

oth2a ______ cans
oth2b ______ cups
oth 2c ______ glasses
oth 2d ______ 300ml bottles
oth 2e ______ 600 ml bottles
oth 2f ______ 1 litre bottles
oth 2g ______ 2 litre bottles

If DON'T KNOW ask:
How many would you usually have on a day like yesterday?

oth3 How much diet soft drink did you drink yesterday? Please say how large the container was in your answer, for example, 375 ml can. (Record didn't drink any as 0 in the first field (cans), don't know as 998 and refused as 999) (Interviewer - prompt for size of can or bottle)

oth 3a ______ cans
oth 3b ______ cups
oth 3c ______ glasses
oth 3d ______ 300ml bottles
oth 3e ______ 600 ml bottles
oth 3f ______ 1 litre bottles
oth 3g ______ 2 litre bottles

If DON'T KNOW ask:
How many would you usually have on a day like yesterday?

oth4 How many cups, glasses, mls or litres of plain water did you drink yesterday? (Record didn't drink any as 0 in the first field (cans), don't know as 998 and refused as 999 and go to beh1)

oth 4a ______ cups
oth 4b ______ glasses
oth 4c ______ mls
oth 4d ______ litres

oth5 Was this water mainly tap water or bottled water? (Single Response)

1. Tap
2. Filtered tap
3. Bottled
998. Can't remember/Don't know
999. Refused

The next section is about more general issues related to food and includes some questions about how you think and act in relation to food.

beh1 How often do you try to avoid eating foods which are high in fat? (Read options. Single Response)

1. Always
2. Often
3. Sometimes
4. Rarely
5. Never
6. Don't know which foods are high in fat

998. Can't remember/Don't know **(Don't read)**
999. Refused **(Don't read)**

beh2 When you use fat or oil in food preparation, how often is it mono or polyunsaturated fat? (Read options. Single Response)

1. Always
2. Often
3. Sometimes
4. Rarely
5. Never
6. Don't use fat or oil
7. I don't understand what these terms mean

998. Can't remember/Don't know **(Don't read)**
999. Refused **(Don't read)**

beh3 When you buy a meal how often do you try to choose healthy foods? (Read options. Single Response)

1. Always
2. Often
3. Sometimes
4. Rarely
5. Never
6. I don't buy meals

998. Can't remember/Don't know **(Don't read)**
999. Refused **(Don't read)**

beh4 If you bought a meal buy from a restaurant, take-away, lunch bar, canteen or other prepared food outlet yesterday, which meal did you buy? (Read options. Single Response)

0. No meals
1. Breakfast
2. Lunch
3. Evening Meal/Dinner
4. Breakfast and lunch
5. Lunch and dinner
6. Breakfast and dinner

998. Can't remember/Don't know **(Don't read)**
999. Refused **(Don't read)**

beh5 Thinking back to the last time that you bought a meal from a restaurant, take-away, lunch bar, canteen or other prepared food outlet, was that meal breakfast, lunch or evening meal? (Read options. Single Response)

1. Breakfast
2. Lunch
3. Evening Meal
4. Don't buy meals out

998. Can't remember/Don't know **(Don't read)**
999. Refused **(Don't read)**

beh6 Thinking about the last meal that you bought from a restaurant, take-away, lunch bar, canteen, were there enough healthy choices available? (Read options. Single Response)

0. No
1. Yes
2. Yes but I didn't want any of them
3. I don't know what a healthy choice is
4. I am uninterested in healthy choices

998. Can't remember/Don't know **(Don't read)**
999. Refused **(Don't read)**

beh7 How important is it for you to have healthy choices available when you buy a meal? (Read options. Single Response)

1. Very important
2. Quite important
3. Neither important nor unimportant
4. Quite unimportant
5. Not at all important

998. Can't remember/Don't know **(Don't read)**
999. Refused **(Don't read)**

Thinking about your neighborhood (within one mile or 1.6km from home), do you agree with the following statements:

beh8 There is a lack of access to adequate food -shopping is a problem. (Read options. Single Response)

1. Strongly agree
2. Agree
3. Neither agree nor disagree
4. Disagree
5. Strongly disagree

998. Can't remember/Don't know **(Don’t read)**
999. Refused **(Don’t read)**

beh9 A large selection of fruits and vegetables are available. (Read options. Single Response)

1. Strongly agree
2. Agree
3. Neither agree nor disagree
4. Disagree
5. Strongly disagree

998. Can't remember/Don't know **(Don’t read)**
999. Refused **(Don’t read)**

beh10 A large selection of healthy take-away foods are available. (Read options. Single Response)

1. Strongly agree
2. Agree
3. Neither agree nor disagree
4. Disagree
5. Strongly disagree

998 Can't remember/Don't know **(Don’t read)**
999 Refused **(Don’t read)**

This next section is about what you currently eat and why you eat what you do.

chg1 In the past 12 months, have you tried to change the amount of fruit you eat? (Single Response. If they say yes and don't specify increase or decrease, probe for that)

0. No **(Go to chg3)**
1. Yes, tried to increase amount
2. Yes, tried to decrease amount **(Go to chg3)**

998. Can't remember/Don't know **(Go to chg3)**
999. Refused **(Go to chg3)**

chg1a What influenced you to try and eat more fruit? Try to think of all the reasons. (Interviewer note: After first responses, PROBE Anything else?)

1. Related to weight/diet
2. To reduce bowel problems/constipation/ improve digestion
3. Improve health in general /health reasons (unspec)
4. To improve fitness / for sports
5. Advice from family/friends / influence of others I cook for
6. Influence of others/person who buys/prepares food is eating more
7. Influence from others/ family-children/encourage family to eat more
8. Advertising (any form) /specific campaigns 2 fruit & 5 veg
9. It's good for you/I wasn't eating enough
10. I like it/ we just like eating fruit / it tastes good

996. Other Specify

Specify other here ____

998. Don't know / no particular reason

999. Refused

chg2 What steps have you taken to try and increase the amount of fruit you eat? Try to think of all the things that you did. (Interviewer note: After first responses, PROBE Anything else?)

1. Trying-buying-eating-using a bigger variety /different types
2. Buying more/ asking my partner to buy-cook more-change our diet
3. Taking fruit to work /uni
4. Having fruit on display at home/trying to have more fruit around
5. Having fruit with-for dessert
6. Eating more making fruit a larger part of diet/eat some every day
7. Substituting /fruit as snack/having fruit for morning tea/lunch

096. Other

Specify other here ____

998. Don't know / no particular step

999. Refused

chg3 Do you feel you eat more, less, or about the same amount of fruit as you did 12 months ago? (Single Response)

1. Eat more now
2. Eat less now
3. Eat about the same now

998. Can't remember/Don't know

999. Refuse

chg4 What are the main things that make it difficult for you to eat more fruit? Try to think of all the things that you think make it difficult (Interviewer note: After first responses, PROBE Anything else?)

1. Nothing / I already eat enough / I can't fit anymore
2. Don't like fruit / lack of interest in fruit / I get bored with it
3. Cost/too expensive
4. Difficulty in changing eating habits / I just don't think of it
5. The time it takes to buy-prepare fruit to eat/no time/ too busy
6. Hard to find good quality
7. Not enough variety in types of fruit available/seasonal availability
8. Doesn't appeal in cold weather/not tempting in winter

096. Other
Specify other here ____
998. Don't know/can't remember
999. Refused

chg5 What do you think is the recommended number of serves of fruit that should be eaten each day? One serve of fruit is equal to one medium piece of fruit or half cup of cooked or canned fruit. (Single Response. Code none as 0, don't know as 998 and refused as 999)

____

chg6 In the past 12 months, have you tried to change the amount of vegetables you eat? (Single Response. If they say yes and don't specify increase or decrease, probe for that)

0. No **(Go to chg9)**
1. Yes, tried to increase amount
2. Yes, tried to decrease amount **(Go to chg9)**

998. Can't remember/Don't know **(Go to chg9)**
999. Refused **(Go to chg9)**

chg7 What influenced you to try and eat more vegetables? Try to think of all the reasons. (Interviewer note: After first responses, PROBE Anything else?)

1. Related to weight/diet
2. To reduce bowel problems/constipation/ improve digestion
3. Improve health in general /health reasons (unspec)
4. To improve fitness / for sports
5. Advice from family/friends / influence of others I cook for
6. Influence of others/person who buys/prepares food is eating more
7. Influence from others/ family-children/encourage family to eat
8. Advertising (any form) /specific campaigns 2 fruit & 5 veg
9. It's good for you/I wasn't eating enough
10. I like it/ we just like eating vegetables /they taste good
11. Substitute for other foods/trying to eat less/no red meat

096. Other
Specify other here ____
998. Don't know / no particular reason
999. Refused

chg8 What steps have you taken to try and increase the amount of vegetables you eat? Try to think of all the things that you did. **(Interviewer note: After first responses, PROBE Anything else?)**

1. Trying-buying-eating-using a bigger variety /different types
2. Buying more/ asking my partner to buy-cook more-change our diet
3. Trying different recipes/vegetarian recipes
4. Cooking more vegetables in each meal
5. Eating more making vegies as a larger part of diet/eat some every day
6. Substituting /veg as snack/having veg for morning tea/lunch reason
7. Cutting down on red meat/substituting with vegetables
8. Growing my own vegetables

096. Other
Specify other here ____
998. Don't know / no particular
999. Refused

chg9 Do you feel you eat more, less, or about the same amount of vegetables as you did 12 months ago? **(Single Response)**

1. Eat more now
2. Eat less now
3. Eat about the same now

998. Can't remember/Don't know
999. Refuse

chg10 What are the main things that make it difficult for you to eat more vegetables? Try to think of all the things that you think make it difficult **(Interviewer note: After first responses, PROBE Anything else?)**

1. Nothing / I already eat enough / I can't fit anymore in
2. Don't like vegetables children don't like
3. Cost/too expensive
4. Difficulty in changing eating habits / I just don't think of it
5. The time it takes to buy-prepare vegies to eat/no time/ too busy
6. The effort it takes to prepare vegies to eat/ I'm not organised enough
7. Hard to find good quality vegetables
8. Not enough variety in types of vegetables available

096. Other
Specify other here____
998. Don't know/can't remember
999. Refused

chg11 What do you think is the recommended number of serves of vegetables that should be eaten each day? One serve of vegetables is equal to one medium potato, half a cup of cooked vegetables or one cup of salad vegetables. **(Single Response. Code none as 0, don't know as 998 and refused as 999)**

____

chg12 In the past 12 months, have you tried to change the total amount of cereal foods such as breakfast cereal, pasta, rice, bread or bread products that you eat? (Single Response. If they say yes and don't specify increase or decrease, probe for that)

0. No **(Go to chg14)**
1. Yes, tried to increase amount
2. Yes, tried to decrease amount
998. Can't remember/Don't know **(Go to chg14)**
999. Refused **(Go to chg14)**

chg13 What influenced you to try to change the amount of breakfast cereal you eat? Try to think of all the reasons. (Interviewer note: After first responses, PROBE Anything else?)

1. Related to weight/diet
2. To reduce bowel problems/constipation/ improve digestion
3. Improve health in general /health reasons (unspec)
4. Improve fitness/more
5. As special diet for medical reasons/doctor/dietitian advised me
6. Getting hungry at work later/not eating breakfast
7. To increase carbohydrates /better balance in my diet in general
8. Advertising / Health Dept campaign/cooking shows on TV
9. To increase my fibre intake
096. Other
Specify other here ____
998. Don't know / no particular
999. Refused

chg131 What influenced you to try to change the amount of the other cereal foods you eat? Try to think of all the reasons. (Interviewer note: After first responses, PROBE Anything else?)

1. Related to weight/diet
2. To reduce bowel problems/constipation/ improve digestion
3. Improve health in general /health reasons (unspec)
4. Improve fitness/more
5. As special diet for medical reasons/doctor/dietitian advised me
6. Getting hungry at work later/not eating breakfast
7. To increase carbohydrates /better balance in my diet in general
8. Advertising / Health Dept campaign/cooking shows on TV
9. To increase my fibre intake
096. Other
Specify other here ____
998. Don't know / no particular reason
999. Refused

chg14 Have you tried to increase the amount of breakfast cereal you eat? (Single Response)

0. No
1. Yes
998. Can't remember/Don't know
999. Refused

chg16 Have you tried to increase the amount of pasta you eat? (Single Response)

0. No
1. Yes
998. Can't remember/Don't know
999. Refused

chg17 Have you tried to increase the amount of rice you eat? (Single Response)

0. No
1. Yes
998. Can't remember/Don't know
999. Refused

chg20 Have you tried to increase the amount of bread you eat? (Single Response)

0. No
1. Yes
998. Can't remember/Don't know
999. Refused

chg24 Do you feel you eat more, less, or about the same amount of cereal foods in total than you did 12 months ago? (Single Response)

1. Eat more now
2. Eat less now
3. Eat about the same now
998. Can't remember/Don't know
999. Refused

chg25 What are the main things that make it difficult for you to eat more cereal foods? Try to think of all the things that you think make it difficult (Interviewer note: After first responses, PROBE Anything else?)

1. Nothing / I already eat enough / I can't fit anymore in
2. Don't like cereal foods/not interested /I get bored with it
3. Cost/too expensive
4. I don't like eating in the morning/don't eat breakfast
5. The time it takes to prepare / time it takes to make fresh muesli
6. The effort it takes to prepare / effort it takes to make fresh muesli
7. I don't have time to eat breakfast/ get out of bed too late
096. Other
Specify other here____
998. Don't know/can't remember
999. Refused

chg26 What do you think is the recommended minimum number of serves of cereal foods that should be eaten each day? One serve of cereal food is equal to one slice of bread, one cup of breakfast cereal or half a cup of cooked rice or pasta. **(Code none as 0, don't know as 998 and refused as 999)**

____

The next section is about fats

chg27 Can you think of any recommended ways of reducing fat in the diet? Try to think of all the ways **(Interviewer note: After first responses, PROBE Anything else?)**

1. Eat less-don't eat fatty take away foods/eat home cooked meals
2. Use less fat/oil in
3. Eat less cakes/biscuits/chocolates/nuts/potato chips/cool drinks
4. Choose lean meat/trim fat from meat/remove skin from chicken
5. Use less butter margarine on
6. Avoid fatty meats (sausages, salami, bacon
7. Switch to low fat milk/cheese/
8. Use low fat cooking methods (Grill, steam, microwave, drain fat)
9. Eat buy more low fat foods (ie bread, cereals, fruit, vegetables, legumes)
10. Eat less red meat/meat in general/shift to white meat
11. Eat less saturated fat/animal fats
12. Choose polyunsaturated fats (polyunsaturated margarine/vegetable oils)
13. Check the fat content in packaged/precooked/processed foods
14. Eat less Trans fats
15. Eat mono unsat fats/olive oil

096. Other
Specify other here ____
998. Don't know/can't remember
999. Refused

chg28 Can you think of any general recommendations about the type of fat you should eat? Try to think of all the recommendations **(Interviewer note: After first responses, PROBE Anything else?)**

1. Eat less saturated fat/animal fats
2. Choose polyunsaturated fats (margarine, vegetable oils)
3. Don't eat any fat/avoid all fats
4. Eat low cholesterol foods
5. Heart Foundation recommendations
6. Omega 3 fats/eat essential fats from fish
7. Eat less Trans
8. Eat mono unsat fats/olive oil

096. Other
Specify other here _______________________________________
998. Don't know/can't remember
999. Refused

chg29 In the past 12 months, have you tried to cut down on the amount of fat and fatty food in your diet? (Single Response)

0. No
1. Yes
998. Can't remember/Don't know
999. Refused

chg31 Do you now feel you eat more, less, or about the same amount of fat and fatty foods as you did 12 months ago?(Single Response)

1. Eat more now
2. Eat less now
3. Eat about the same now
998. Can't remember/Don't know
999. Refused

chg32 In the past 12 months, have you tried to change the types of fat you include in your diet? (Single Response)

0. No
1. Yes
998. Can't remember/Don't know
999. Refused

chg34 Are you trying to cut down the amount of fat that you eat? (Single Response)

0. No
1. Yes
998. Can't remember/Don't know
999. Refused

chg33 What steps have you taken to try and decrease the amount of fat you eat or change the type of fat you eat? Try to think of all the things that you did. (Interviewer note: After first responses, PROBE Anything else?)

1. Just by eating less (not specific)
2. Not eating/using/cut down butter/margarine
3. Avoid certain foods (Like bacon/fatty meats/pies, pasties/sausage rolls)
4. Cutting down on red meat/reduced meat in my diet/became vegetarian
5. Avoid/eat less ice cream/cakes/biscuits/chocolates/nuts/potato chips
6. Eating less fast foods/no/less takeaways
7. Reducing dairy products/milk, cheese
8. Changing to unsaturated (poly, mono) margarine
9. Changing from polyunsaturated fats to monounsaturated fats
10. Eating low-fat products
11. Eating fruit and vegies instead on fatty foods
12. Eating more fish
13. Eating other leaner meats in general/chicken (not fish)
14. Don't fry/bake or grill instead/ steam vegies
15. Minimum/no oil/fat/ cream in cooking/cutting down on oil
16. Trim fat off meat/skin off chicken
17. Changing to olive oil/canola/grape seed/ Pure and simple
18. Buying foods with lower fat content/substituting fatty foods
19. Avoiding trans fats

096. Other
Specify other here____
998. Don't know/can't remember
999. Refused

chg35 You said you are either trying to cut down the amount, or change the type of fat that you eat. What influenced you to change either the amount or type of fat that you eat? Try to think of all the things that influenced you. (Interviewer note: After first responses, PROBE Anything else?)

1. To lose/control weight
2. To reduce the risk of heart disease
3. To reduce cholesterol levels
4. Special diet for medical reasons
5. To improve health in general
6. To improve fitness
7. Advice from family and friends
8. Advertising/media/Heart Foundation/Health Dept
9. Education/became aware of fat in diet/reading

096. Other
Specify other here ____
998. Don't know/can't remember
999. Refused

chg36 What are the main things that make it difficult for you to avoid fat and fatty foods? Try to think of all the things that you think make it difficult (Interviewer note: After first responses, PROBE Anything else?)

1. Nothing
2. Person buying/preparing my food buys/uses fat and fatty foods
3. Foods I enjoy tend to be high in fat/fatty food is tempting
4. Difficult to change eating habits/families habits
5. The time it takes to prepare/shop for low fat foods
6. The effort it takes to prepare low fat food/I'm not organised
7. Low fat foods not readily available at lunchtime or when eating out
8. I eat out/ have takeaways a lot

096. Other
Specify other here ____
998. Don't know/can't remember
999. Refused

chg37 In the past 12 months have you tried to change the amount of calcium-rich foods you eat? (Single Response)

0. No
1. Yes, tried to increase amount
2. Yes, tried to decrease amount

998. Can't remember/Don't know
999. Refused

The next section is about body weight and how you feel about it and your diet. It is important to get a picture of all the different groups in our community. (For Women Only) However if you are pregnant, then we won't ask about your weight at this time.

Are you pregnant?

0. No
1. Yes **(Go to hth1)**

bod1 What is your height without shoes? (Single Response. Code Unsure/Don't Know/Can't remember as 998 and Refused as 999 in FIRST field.)

bod1a Centimetres ___ OR

bod1b Feet ___ **bod1c** Inches ___

bod2 How much do you weigh without clothes or shoes? (Single Response. Code Unsure/Don't Know/Can't remember as 998 and Refused as 999 in FIRST field)

bod2a Kilograms (Kg) ____ OR

bod2b Stones ____ **bod2c** Pounds ____

bod3 What is your waist circumference? (Interviewer note: You should measure the circumference of your waist at navel level against your skin. It is important that you don't suck your stomach in or push it out, but stand completely relaxed when you take the measurement) (Single Response. Code Unsure/Don't Know/Can't remember as 998 and Refused as 999 in FIRST field.)

bod3a Centimetres ____ **bod3b** Inches ____

For men use the below if waist circumference is not available. Enter 0 if not used

Pant size _______ cm **bod3c** ________ins **bod3d**

bod4 Do you consider yourself to be? (Read Options. Single Response.)

1. Underweight
2. About right
3. Somewhat overweight
4. Very overweight
998. Unsure/don't know/Can't remember
999. Refused

bod5 In the past 12 months, have you been trying to? (Read Options. Single Response.)

1. Lose weight
2. Gain weight
3. Neither
998. Unsure/don't know/Can't remember
999. Refused

bod6 What influenced you to try to lose/gain weight? Try to think of all the things that influenced you. (Interviewer note: After first responses, PROBE Anything else?)

1. To reduce risk of heart disease
2. To improve health in general/want to get pregnant
3. To improve fitness/I felt tired sluggish/short of breath
4. To look better
5. My clothes wouldn't fit
6. Had put weight on/I felt fat/I was overweight
7. Advice from family and friends
8. Feeling uncomfortable (unspecified)/general discomfort/to feel better
9. Advice from a doctor/health professional
10. An advertising campaign
096. Other
What other influence was that? ____
998. Don't know/can't remember
999. Refused

bod7 What steps have you taken to try to lose or gain weight? Try to think of all the things that you did. (Code don't know as 998 and refused as 999 in bod7a and go to bod8) (Interviewer note: After first responses, PROBE Anything else?)

1. Nothing
2. More exercise/walking more/play tennis/more activity
3. Changed diet/eating habits(unspecified)/went on diet
4. Began to be concerned about nutrition/began to eat more balanced diet
5. Eat smaller portions in general/eat less/less food intake
6. Eat more/a lot more /quantity
7. Eat regularly and often
8. Eat lots of eggs/steaks/similar foods
9. Cut out fat(saturated, animal fats)/less butter/ cut down on fatty food
10. High carbohydrate diet
11. Eating more fruit and vegies
12. Stopped snacking between meals/cut down on snacking
13. Stopped eating junk food/cut down take aways/fast food
14. Joined weight watchers/a diet place/Easy slim weight loss
15. Eat smaller/avoid high calorie food /chocolate, sweets
16. Asked a Dietitian/doctor/other health professional for help
17. Joined a Gym
18. Eating a low carb diet
19. Eating a high protein diet
20. Using meal replacement drinks
21. Using medication /diet pills/ suppressants
22. Had surgery (gastric banding, bypass, stapling, liposuction

096. Other
Specify other here ____
998. Don’t know/can’t remember
999. Refused

bod8 And how does your current weight compare with your weight 12 months ago? Do you (Read options. Single response)

1. Weight more
2. Weight less
3. Weigh about the same

998. Can't remember/Don't know
999. Refused

bod10 What are the main things that make it difficult for you to control your weight? Try to think of all the things that you think make it difficult (Code don't know as 998 and refused as 999 in bod10a and go to bod10)(Interviewer note: After first responses, PROBE Anything else?)

1. Nothing
2. Difficult to change eating habits
3. Enjoy my food /food helps me get through
4. Don't like exercise
5. No time to exercise /work long hours
6. Eat out regularly/have take-aways
7. No will power to eat better
8. No will power to exercise
9. I am a shift worker/long hours I work/work commitments
10. I don't exercise (unspecified whether time or inclination)
11. I like fattening /sweet food/I have a sweet tooth
12. Medical problems (unspecified)
13. I like my alcohol/beer/wine/friends convince me to drink
14. No time to shop for healthy food
15. No time to cook
16. I eat what I am given/what the family eats

096. Other
Specify other here ____
998. Don’t know/can’t remember
999. Refused

Now I am going to ask you about your perception about what you eat and how it might affect your health.

hth1 What health problems do you think are associated eating too much fat or fatty foods? (Interviewer note: After first responses, PROBE Anything else?)

1. Doesn't cause health problems
2. Cancer (unspecified)
3. Circulation problems (unspecified)
4. Heart disease/heart attack/heart problems
5. Artherosclerosis/hardening/blocked arteries
6. High blood pressure/hypertension/blood pressure
7. Stroke
8. Diabetes/high blood sugar/sugar problems
9. High cholesterol
10. Obesity/gaining weight/overweight
11. Lethargy/low energy/fatigue/low stamina/tired/run down/sluggish
12. Unfit/lack of fitness
13. Bowel/Colon Cancer

096. Other
Specify other here ____
998. Don’t know/can’t remember
999. Refused

hth2 What health problems do you think are associated with not eating enough fruit and vegetables? Try and think of all the possible health problems (Code don't know as 998 and refused as 999 in hth2a and go to hth3) (Interviewer note: After first responses, PROBE Anything else?)

1. Doesn't cause health problems
2. Bowel cancer/colon cancer
3. Heart disease/heart attack/heart problems
4. Constipation/poor irregular bowel movements/lack of regularity
5. Digestion problems (unspecified)/acid reflux
6. Intestinal disorders/bowel problems/obstructions/diverticulosis
7. Skin problems (inc unspecified)/bad skin/acne
8. Scurvy/beri beri/rickets
9. Obesity/gaining weight/overweight
10. Vitamin & mineral deficiencies/lack of nutrition (unspecified)
11. Poor immunity/prone to colds/flu/low resistance to disease
12. Anaemia/iron deficiency/lack of iron
13. Not enough fibre-roughage
14. Lethargy/low energy/fatigue/low stamina/tired/run down/sluggish
15. General health problems/unwell/sick/run down
16. Cancer (Unspecified)
17. Diabetes

096. Other
Specify other here ____
998. Don't know/can't remember
999. Refused

hth3 What health problems do you think are associated with not eating enough cereal food or bread. Try and think of all the possible health problems (Interviewer note: After first responses, PROBE Anything else?)

1. Doesn't cause health
2. Bowel cancer/colon cancer
3. Heart disease/heart attack/heart problems
4. Constipation/poor irregular bowel movements/lack of regularity
5. Digestion problems (unspecified)/acid reflux
6. Intestinal disorders/bowel problems/ obstructions/diverticulosis
7. Vitamin & mineral deficiencies/lack of nutrition (unspecified)
8. Not enough fibre-roughage
9. Lethargy/low energy/fatigue/low stamina/tired/run down/sluggish
10. General health problems/unwell/sick/run down
11. Obesity/weight gain
12. Cancer unspecified
13. Diabetes

096. Other
What other health problem was that? _________________________________
998. Don't know/can't remember
999. Refused

hth4 What health problems do you think are associated being overweight? Try and think of all the possible health problems (Interviewer note: After first responses, PROBE Anything else?)

1. Doesn't cause health problems
2. Circulation problems (unspecified)
3. Heart disease/heart attack/heart problems
4. High blood pressure/hypertension/blood pressure
5. Respiratory problems/ /breathing problems (unspecified)
6. Joint problems/knee problems
7. Back problems
8. Strain on muscular/skeletal system/immobility/not walking well
9. High cholesterol
10. Obesity/gaining weight/overweight
11. Low self-esteem/depression/feel bad/horrible/uncomfortable
12. Lethargy/low energy/fatigue/low stamina/tired/run down/sluggish
13. Unfit/lack of fitness
14. General health problems/unwell/sick/run down
15. Cancer unspecified
16. Bowel/Colon cancer
17. Diabetes

096. Other
Specify other here ____
998. Don’t know/can’t remember
999. Refused

Now I am going to ask you what you think about breastfeeding babies.

brst1 How important do you think it is for mothers to breast feed their babies. Do you think it is: (Read options. Single response)

1. Not at all important
2. Not very important
3. Neither important nor unimportant
4. Somewhat important
5. Very important

998. Can't remember/Don't know
999. Refuse

brst2 What do you think are the benefits of breast-feeding for babies? Try to think of all the possible benefits (Interviewer note: After first responses, PROBE Anything else?)

1. Immunity
2. Vitamins & minerals/nutrition
3. Tailor made/ perfect food
4. Health benefits/good for baby (unspec)
5. No chemicals/natural
6. Convenience/easier
7. Bonding/security/love/emotional
8. Better development/intelligence

096. Other

Specify other here ____

998. Don't know/can't remember

999. Refused

brst2b What do you think are the benefits of breast-feeding for mothers? Try to think of all the possible benefits (Interviewer note: After first responses, PROBE Anything else?)

1. Weight loss/get to pre-pregnancy weight
2. Prevents
3. Prevents diabetes
4. Convenience/easier
5. Bonding/security/love/emotional

096. Other

Specify other here ____

998. Don't know/can't remember

999. Refused

brst3 For how long do you think mothers should breast-feed their babies? (Code don't think they should breast feed as 0, as long as possible as 997 (See interviewer note) don't know as 998 and refused as 999)

______days

______ months

______years

Interviewer note: If respondent says as long as possible ask for an estimated time.

brst3a For how long do you think mothers should breast-feed their babies?

______days

______ months

______years

brst3b For how long do you think it is important for babies to be exclusively breastfed without any other fluids, including water and infant formula, or any solid foods? (Code don't think they should breast feed as 0, don't know as 998 and refused as 999)

______days
______ months
______years

Interviewer note: If respondent says as long as possible ask for an estimated time.

brst3b For how long do you think it is important for babies to be exclusively breastfed without any other fluids, including water and infant formula, or any solid foods?

______days
______ months
______years

brst4 What do you think make it difficult for women to continue to breast-feed their babies for at least six months? Try to think of all the things that might make it difficult. (Interviewer note: After first responses, PROBE Anything else?)

1. Need to return to work
2. Poor supply of breast milk
3. Problems with breast feeding (eg. sore nipples
4. Convenience of bottle/ inconvenience
5. No public acceptance
6. Stress/pressure/no time
7. Don't like breastfeeding/don't like doing it/embarrassing
8. Don't think there is a problem

096. Other
Specify other here ____
998. Don't know/can't remember
999. Refused

brst5 What do you think would make it easier for women to continue to breast-feed their babies for at least six months? Try to think of all the things that might make it easier. (Interviewer note: After first responses, PROBE Anything else?)

1. Not having to work
2. Lack of stress/pressure/more time
3. More breast feeding facilities
4. Education about breast
5. Information about diet & nutrition to keep up supply
6. Support/encouragement and cooperation from husband
7. Public acceptance/community support/change attitudes
8. Don't think that there is a problem

096. Other
Specify other here ______________________________________
998. Don't know/can't remember
999. Refused

In which of these places do you think that it is acceptable for a woman to breastfeed her baby (Read options. Single Response)

brst6 Shopping Centres

0. No
1. Yes
2. Depends on how obvious it was
998. Can't remember/Don't know **(Don't read)**
999. Refused **(Don't read)**

brst7 Workplaces

0. No
1. Yes
2. Depends on how obvious it was
998. Can't remember/Don't know **(Don't read)**
999. Refused **(Don't read)**

brst8 Restaurants

0. No
1. Yes
2. Depends on how obvious it was
998. Can't remember/Don't know **(Don't read)**
999. Refused **(Don't read)**

brst9 On public transport (buses and trains)

0. No
1. Yes
2. Depends on how obvious it was
998. Can't remember/Don't know **(Don't read)**
999. Refused **(Don't read)**

brst10 Would it be acceptable if a room/space was provided for women to breast feed their babies in any of these public places?

0. No
1. Yes
2. Depends on how obvious it was
998. Can't remember/Don't know **(Don't read)**
999. Refused **(Don't read)**

Now some more general questions about where you get your information on food and nutrition.

inf1 In the last 12 months, what have been your main sources of nutrition and dietary information? (Code don't know as 998 and refused as 999 in inf1a and go to inf2) (Interviewer note: After first responses, PROBE Anything else?)

1. Books
2. Community Health Centres
3. Dietitians
4. Doctors
5. Family and friends
6. Food manufacturers
7. Health Department
8. Health Food Shops
9. Magazine articles
10. National Heart Foundation/Cancer Council/NGO
11. Newspaper articles
12. Radio interviews
13. Schools
14. TV ads
15. TV programs
16. Nurses/Health workers
17. Internet/website
18. Naturopath/other alternative practitioners
19. Pharmacy
20. Pamphlets/

096. Other
Specify ________________________________

998. Don't know/Can't remember
999. Refused

Part 3- Support for Government Nutrition Policies/Activities

In your opinion, how important is it that governments financially support the following nutrition activities? For each, please say whether it is very important, quite important neither important nor unimportant, not very important or not at all important.

act1 Research investigating what Western Australians are eating and how their diet is changing over time (Read options. Single Response)

1. Very important
2. Quite important
3. Neither important nor unimportant
4. Not very important
5. Not at all important

998. Can't remember/Don't know **(Don't read)**
999. Refused **(Don't read)**

act2 Monitoring the quality of the food supply (Read options. Single Response)

1. Very important
2. Quite important
3. Neither important nor unimportant
4. Not very important
5. Not at all important
998. Can't remember/Don't know **(Don’t read)**
999. Refused **(Don’t read)**

act3 Providing nutrition publications for the general public (Read options. Single Response)

1. Very important
2. Quite important
3. Neither important nor unimportant
4. Not very important
5. Not at all important
998. Can't remember/Don't know **(Don’t read)**
999. Refused **(Don’t read)**

act4 Advertising campaigns to promote healthy eating to the public (Read options. Single Response)

1. Very important
2. Quite important
3. Neither important nor unimportant
4. Not very important
5. Not at all important
998. Can't remember/Don't know **(Don’t read)**
999. Refused **(Don’t read)**

act5 Working with the food industry to increase the availability of healthy foods (Read options. Single Response)

1. Very important
2. Quite important
3. Neither important nor unimportant
4. Not very important
5. Not at all important
998. Can't remember/Don't know **(Don’t read)**
999. Refused **(Don’t read)**

act6 Are there any other activities related to food and nutrition that governments should financially support? (Read options. Single Response)

0. No
1. Yes
998. Can't remember/Don't know
999. Refused

act6a Specify activity one

____

act6a1 How important would you say it is that the government financially support this activity **(Read options. Single Response)**

1. Very important
2. Quite important
3. Neither important nor unimportant
4. Not very important
5. Not at all important

998. Can't remember/Don't know **(Don't read)**
999. Refused **(Don't read)**

act6b Are there any other activities related to food and nutrition that governments should financially support? **(Read options. Single Response)**

0. No
1. Yes

998. Can't remember/Don't know
999. Refused

act6b Specify Activity two

____

act6b1 How important would you say it is that the government financially support this activity **(Read options. Single Response)**

1. Very important
2. Quite important
3. Neither important nor unimportant
4. Not very important
5. Not at all important

998. Can't remember/Don't know **(Don't read)**
999. Refused **(Don't read)**

act6c Are there any other activities related to food and nutrition that governments should financially support? **(Read options. Single Response)**

0. No
1. Yes

998. Can't remember/Don't know
999. Refused

act6c Specify Activity three

____

act6c1 How important would you say it is that the government financially support this activity (Read options. Single Response)

1. Very important
2. Quite important
3. Neither important nor unimportant
4. Not very important
5. Not at all important

998. Can't remember/Don't know **(Don’t read)**
999. Refused **(Don’t read)**

actd Are there any other activities related to food and nutrition that governments should financially support? (Read options. Single Response)

0. No
1. Yes

998. Can't remember/Don't know
999. Refused

act6d Specify Activity four

____

act6d1 How important would you say it is that the government financially support this activity (Read options. Single Response)

1. Very important
2. Quite important
3. Neither important nor unimportant
4. Not very important
5. Not at all important

998. Can't remember/Don't know **(Don’t read)**
999. Refused **(Don’t read)**

act7a I am going to read out 5 potential Government community education program topics. I want you to rank them for 1 - should be given the highest priority to 5 - should be given the lowest priority. Listen to the list first and then I will repeat the list and ask you to rank it in terms of priority. (Read list twice, the first time without asking for the ranking and the second time asking for the ranking.)

Fat, Fruit and vegetables, Bread and cereals, Weight and weight control, Sugar

Which topic would you give the highest ranking in terms of priority (Read options. Single Response)

1. Fat
2. Fruit and vegetables
3. Bread and cereals
4. Weight and weight control
5. Sugar
998. Can't remember/Don't know
999. Refused

And which topic would be second highest in terms or priority (Read options. Single Response)

1. Fat
2. Fruit and vegetables
3. Bread and cereals
4. Weight and weight control
5. Sugar
998. Can't remember/Don't know
999. Refused

And which topic would be third highest in terms or priority? (Read options. Single Response)

1. Fat
2. Fruit and vegetables
3. Bread and cereals
4. Weight and weight control
5. Sugar
998. Can't remember/Don't know
999. Refused

And which topic would be fourth highest in terms or priority? (Read options. Single Response)

1. Fat
2. Fruit and vegetables
3. Bread and cereals
4. Weight and weight control
5. Sugar
998. Can't remember/Don't know
999. Refused

So just to confirm (READ OUT CODE BELOW) will be the lowest priority? (Enter code to confirm)

1. Fat
2. Fruit and vegetables
3. Bread and cereals
4. Weight and weight control
5. Sugar
997. Incorrect- re-ask ranking question.

act7 Are there any other areas of nutritional or nutrition-related information that you feel ought to be given equal or higher priority than these areas? **(Read options. Single Response)**

0. No
1. Yes
998. Can't remember/Don't know
999. Refused

act7 Specify other priority here **(Single Response)**

____

act7 What rank would you give this other priority? **(Single Response)**

______________+______

act7 Are there any other activities related to food and nutrition that governments should financially support? **(Read options. Single Response)**

0. No
1. Yes
998. Can't remember/Don't know
999. Refused

act7 Specify other priority here

____

act7 What rank would you give this other priority? **(Single Response)**

____

How important would you say it is that the government has control over or regulates the following:

act8a The supply of environmentally friendly food **(Read options. Single Response)**

1. Very important
2. Quite important
3. Neither important nor unimportant
4. Not very important
5. Not at all important
998. Can't remember/Don't know **(Don't read)**
999. Refused **(Don't read)**

act8b A health rating on food labels (Read options. Single Response)

1. Very important
2. Quite important
3. Neither important nor unimportant
4. Not very important
5. Not at all important

998. Can't remember/Don't know **(Don’t read)**
999. Refused **(Don’t read)**

act8c Genetically modified foods (Read options. Single Response)

1. Very important
2. Quite important
3. Neither important nor unimportant
4. Not very important
5. Not at all important

998. Can't remember/Don't know **(Don’t read)**
999. Refused **(Don’t read)**

act8d Food advertising (Read options. Single Response)

1. Very important
2. Quite important
3. Neither important nor unimportant
4. Not very important
5. Not at all important

998. Can't remember/Don't know **(Don’t read)**
999. Refused **(Don’t read)**

act8e Nutrition information on food labels (Read options. Single Response)

1. Very important
2. Quite important
3. Neither important nor unimportant
4. Not very important
5. Not at all important

998. Can't remember/Don't know **(Don’t read)**
999. Refused **(Don’t read)**

act8f Are there any other areas of food or nutrition related activities that you feel ought to be controlled by the government and given equal or higher priority than these areas? (Read options. Single Response)

0. No
1. Yes

998. Can't remember/Don't know
999. Refused

act8f1 What other area?

____

act8g Are there any other areas of food or nutrition related activities that you feel ought to be controlled by the government and given equal or higher priority than these areas? (Read options. Single Response)

0. No
1. Yes
998. Can't remember/Don't know
999. Refused

act8g1 What other area

____

act9 Which of the following would make it easier for you or your family to eat a healthy diet? (Read options. Single Response)

1. Cheaper healthy foods (Single Response)

0. No
1. Yes
998. Can't remember/Don't know
999. Refused

2. More healthy food options provided in take-away and fast food outlets (Single Response)

0. No
1. Yes
998. Can't remember/Don't know
999. Refused

3. Knowing more ways of preparing healthy foods (Single Response)

0. No
1. Yes
998. Can't remember/Don't know
999. Refused

4. Knowing quicker ways of preparing healthy foods (Single Response)

0. No
1. Yes
998. Can't remember/Don't know
999. Refused

5. If I had more information to help me decide which foods are healthy (Single Response)

0. No
1. Yes
998. Can't remember/Don't know
999. Refused

6. If I knew more about cooking (Single Response)

0. No
1. Yes
998. Can't remember/Don't know
999. Refused

7. If my family/partner enjoyed healthy foods (Single Response)

0. No
1. Yes
998. Can't remember/Don't know
999. Refused

8. If could buy more healthy food snacks (Single Response)

0. No
1. Yes
998. Can't remember/Don't know
999. Refused

9. If healthier food were easier to find in supermarkets (Single Response)

0. No
1. Yes
998. Can't remember/Don't know
999. Refused

10. If there was detailed and easy to understand information on food labels (Single Response)

0. No
1. Yes
998. Can't remember/Don't know
999. Refused

11. If all unhealthy foods had a coloured symbol on the label (Single Response)

0. No
1. Yes
998. Can't remember/Don't know
999. Refused

act20 Are there other activities which would make it easier for you and your to eat a healthy diet? (Read options. Single Response)

0. No
1. Yes
998. Can't remember/Don't know
999. Refused

act20a Specify other option here ____

On a scale of very concerned to not at all concerned, how concerned would you say you are about the following:

act21 The sale of high caffeine drinks to children 12 years and younger (Read options. Single Response)

1. Not very concerned
2. Somewhat concerned
3. Neither unconcerned nor concerned
4. Quite concerned
5. Very concerned
998. Can't remember/Don't know **(Don’t read)**
999. Refused **(Don’t read)**

act22 The health effects of eating genetically modified foods (Read options. Single Response)

1. Not very concerned
2. Somewhat concerned
3. Neither unconcerned nor concerned
4. Quite concerned
5. Very concerned
998. Can't remember/Don't know **(Don’t read)**
999. Refused **(Don’t read)**

act23 The amount of fat in foods (Read options. Single Response)

1. Not very concerned
2. Somewhat concerned
3. Neither unconcerned nor concerned
4. Quite concerned
5. Very concerned
998. Can't remember/Don't know **(Don’t read)**
999. Refused **(Don’t read)**

act24 The amount of salt in foods (Read options. Single Response)

1. Not very concerned
2. Somewhat concerned
3. Neither unconcerned nor concerned
4. Quite concerned
5. Very concerned
998. Can't remember/Don't know **(Don't read)**
999. Refused **(Don't read)**

act25 The increasing portion size of foods (Read options. Single Response)

1. Not very concerned
2. Somewhat concerned
3. Neither unconcerned nor concerned
4. Quite concerned
5. Very concerned
998. Can't remember/Don't know **(Don't read)**
999. Refused **(Don't read)**

act26 The effect of the environment on the future of food supplies (Read options. Single Response)

1. Not very concerned
2. Somewhat concerned
3. Neither unconcerned nor concerned
4. Quite concerned
5. Very concerned
998. Can't remember/Don't know **(Don't read)**
999. Refused **(Don't read)**

act27 School canteens should restrict sales of high fat foods such as pies, chips, sausage rolls and fatty snack foods. Do you: (Read options. Single Response)

1. Strongly Agree
2. Agree Slightly
3. Neither agree nor disagree
4. Disagree Slightly
5. Disagree Strongly
998. Can't remember/Don't know **(Don't read)**
999. Refused **(Don't read)**

act27 Who do you think should decide which types of foods are sold in school canteens? (DO NOT READ. Multiple Response. Code don't know as 998 and refused as 999 and go to act28)

1. Children
2. Parents
3. Teachers/principal of school
4. Education Dept
5. Canteen Manager/ Manageress
6. Health Dept
096. Other
Specify other here ____
998. Don’t know
999. Refused

act28 In the last 12 months, has anyone in your household eaten less than they should because you couldn't afford enough food? (Single Response)

0. No
1. Yes
998. Can't remember/Don't know
999. Refused

act29 In the last six months, have you experienced vomiting and/or diarrhoea, which you suspect, may have been food poisoning? (Single Response)

0. No **(Go to food1)**
1. Yes
998. Can't remember/Don't know **(Go to food1)**
999. Refused **(Go to food1)**

act30 Was food poisoning positively identified by a doctor or nurse? (Single Response)

0. No
1. Yes
998. Can't remember/Don't know
999. Refused

These next few questions are about how food is handled in your household.

food1 Do you have any responsibility for doing the food shopping in your household? (Single Response)

0. No
1. Sole responsibility
2. Shared responsibility
998. Unsure/Don’t Know/Can’t Remember
999. Refused

food2 How often do you put your cold or frozen food in an esky to transport it from the shops to home? **(Read options. Single Response)**

1. Always
2. Usually
3. Sometimes
4. Usually not
5. Never
998. Unsure/Don't Know/Can't Remember
999. Refused

food3 Do you have any responsibility for choosing and preparing meals in your household? **(Single Response)**

0. No
1. Sole responsibility
2. Shared responsibility
998. Unsure/Don't Know/Can't Remember
999. Refused

food4 What do you think is the maximum temperature a fridge should operate at to keep food in good condition **(Single Response. Record don't know as 998 and refused as 999)**

___________Degrees Celsius

food5 Do you have a thermometer in your main fridge at home? **(Single Response)**

0. No
1. Yes
2. Don't have fridge
998. Unsure/Don't Know/Can't Remember
999. Refused

Part 4- Demographics

Finally a few questions about you. These will help us to understand the answers we get in this survey

cha1 What is the highest level of primary or high school that you have completed? **(Single Response. Interviewer note: Prompt if necessary)**

1. Never attended school
2. Currently still at school
3. Year 8 or below
4. Year 9 or equivalent
5. Year 10 or equivalent
6. Year 11 or equivalent
7. Year 12 or equivalent (matriculation/leaving)
998 Unsure/Don't Know/Can't Remember
999 Refused

cha2 Have you completed any qualifications (since leaving school)? (Single Response. Interviewer note: Prompt if necessary)

0. No **(Go to cha4)**
1. Yes
998 Unsure/Don't know/Can't remember **(Go to cha4)**
999 Refused **(Go to cha4)**

cha3 What is the highest qualification you have completed? (Single Response. Interviewer note: Prompt if necessary)

1. Bachelor degree or higher
2. Diploma or certificate taking more than 12 months full time
3. Diploma or certificate taking less than 12 months full time
4. Trade / apprenticeship
998. Unsure/Don't know/Can't remember
999. Refused

cha4 Which geographic area were you born in? (Single Response*)*

1. Australia
2. Eire and UK including Wales, Scotland and Northern Ireland
3. New Zealand
4. North America
5. Central and South America
6. Africa
7. Europe
8. Middle East
9. ASEAN Country (These include Burma, Brunei Darussalam, Cambodia, Indonesia, Laos, Malaysia, Philippines, Singapore, Thailand and Vietnam)
10. China
11. India
096. Other
Other geographic area were you born in specified? ___________________________
998. Unsure/Don't know/Can't remember
999. Refused

cha5 In what year did you first arrive in Australia to live? (Single Response)

____

cha6 Are you of Aboriginal or Torres Strait Islander origin? (Single Response)

0. No
1. Yes, Aboriginal
2. Yes, TSI
3. Yes, both Aboriginal and TSI
998. Unsure/Don't know/Can't remember
999. Refused

cha7 Which ONE of the following best describes your current employment status? Are you: **(Read options. Single Response. Interviewer note: This question relates to MAIN occupation. A full-time student who works part-time is coded as a student)**

1. Self employed
2. Employed for wages, salary or payment-in-kind
3. Unemployed for less than one year
4. Unemployed for more than one year
5. Engaged in home duties
6. Retired
7. Unable to work
8. A student

096. Other
998 Unsure/Don't Know/Can't Remember
999 Refused

cha8 What best describes your current living arrangements? **(Read Options. Single Response)**

1. Living with my parent(s)
2. Living with other family members
3. Living with friends
4. Living with a partner and children
5. Living with a partner but no children
6. Living alone
7. Living in a nursing home
8. Living in a retirement village

096. Other living arrangements
Other living arrangements specified ________________________________
998 Unsure/Don't Know/Can't Remember
999 Refused

cha9 Which best describes your household money situation? **(Read Options. Single Response)**

1. I am / we are spending more money than I / we get
2. I / we have just enough money to get us through to the next pay day
3. There's some money left over but I / we just spend it
4. I / we can save a bit every now and then
5. I / we can save regularly
6. I / we can save a lot

998. Unsure/Don't know/Can't remember
999. Refused

cha10 I would now like to ask you about your household's income. We are interested in how income relates to health, lifestyle and access to food. Before tax is taken out, which of the following ranges best describes your household's income, from all sources, over the past 12 months? **(Read Options. Single Response)**

1. Under $20,000
2. $20,000 - $40,000
3. $40,000 - $60,000
4. $60,000 - $80,000
5. $80,000 - $100,000
6. $100,000 - $120,000
7. $120,000 - $140,000
8. More than $140,000

998. Unsure/Don't Know/Can't Remember
999. Refused

Now I would like to ask you a few questions that we use to ensure that the information we collect is representative of the whole of WA.

cha12 Including yourself, how many people aged 18 years to 64 usually live in this household? **(Record refused as 999)**

____

cha13 How many listings do you have in the White Pages telephone book? **(Record don't know = 998, refused = 999)**

____

That is the end of the interview.

Thank you for your time. Just to remind you my name is________ on behalf of the Department of Health.

If you have any questions about this research you can telephone our office on XXXXXXXXX

I certify that this is a true, accurate and complete interview, conducted in accordance with IQCA standards and the ICC/ESOMAR International Code of conduct. I will not disclose to any other person the content of this questionnaire or any other information relating to this project.

[Interview - please initial questionnaire]

Initials - __________
